# Controlled Peptide Capture and Release in 3D‐Printed Multimaterial Microstructures

**DOI:** 10.1002/advs.76505

**Published:** 2026-07-16

**Authors:** Niklas Schwegler, Thomas Heim, Philipp Mainik, Eva Blasco, Franziska Thomas

**Affiliations:** ^1^ Institute of Organic Chemistry Heidelberg University Heidelberg Germany; ^2^ Institute for Molecular Systems Engineering and Advanced Materials Heidelberg University Heidelberg Germany

**Keywords:** 3D printing, coiled‐coils, hydrogels, peptides, stimuli‐responsive materials, two‐photon laser printing

## Abstract

High‐resolution two‐photon laser printing has revolutionized the fabrication of complex 3D micro‐ and nanostructures across a wide range of materials. However, the implementation of engineered biomolecules as functional, stimuli‐responsive units remains underexplored. In this study, we present a method for fabricating 3D‐printed hydrogel microstructures that contain de novo designed heterodimeric coiled‐coil peptides to allow controlled peptide capture and release. Covalent incorporation of one coil strand into the 3D‐printed network was combined with fluorescent labelling of the complementary strand. Controlling the assembly and disassembly of the coiled coil enabled selective binding and subsequent programmable release of the fluorescently labelled peptide using various external stimuli, such as pH, ionic strength, temperature, or peptide competitors. Using a set of orthogonal coiled‐coil peptides, we fabricated multimaterial microstructures in which spatially resolved coiled‐coil functionalization was achieved. Under complete spatiotemporal control, it was shown that the complementary coil strands could be captured and released selectively. This work demonstrates the use of discretely folded, chemically synthesizable peptides for 2PLP fabrication for the first time. Utilizing coiled‐coil interactions as molecular handles enables the production of stimuli‐responsive and reconfigurable hydrogel microstructures. This approach opens up possibilities for dynamic biomaterials, programmable drug delivery, and biochemical process engineering at the microscale.

## Graphical abstract

Two‐photon laser printing of 3D microstructures using a peptide‐containing ink is shown. The microstructures allow for controlled capture and release of target peptides through coiled‐coil interaction with the printed peptide inside the material. Based on these properties, orthogonal capture and stimuli‐mediated release possibilities inside printed multimaterial structures are presented, allowing for spatiotemporal control of multiple functionalities inside one structure.

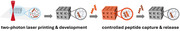

## 1 Introduction

Recent progress in additive manufacturing has significantly broadened the landscape of achievable material architectures and functionalities [1, 2]. For soft materials with biological relevance, the demand for highly controlled, digitally defined 3D architectures can be effectively addressed through advanced photopolymerization‐based additive manufacturing. Among these approaches, two‐photon laser printing (2PLP) stands out for its exceptional resolution and structural precision [3, 4, 5]. Current research focuses on exploring intelligent materials that can respond to specific stimuli in their environment. By changing external parameters (e.g., temperature, pH, or solvent), effects like controlled volume or shape changes can be achieved [3, 6, 7, 8]. While the field of stimuli‐responsive 3D‐printed materials has expanded vastly, the use of functional biomolecules such as peptides and proteins as the intelligent, responsive unit is underrepresented. Most studies including proteins or peptides focus on the utilization of simple sequence‐specific functional peptides (e.g., cell‐adhesive RGD peptides) or macromolecular assemblies as structural material components (e.g., collagen‐based networks) [9, 10, 11, 12, 13, 14, 15]. However, it is the class of discretely folded functional peptides and proteins that shows a variety of highly specific responsive behaviors at mild, biologically relevant conditions. Through manipulation of supramolecular peptide‐peptide and peptide‐solvent interactions (hydrogen bonding, hydrophobic effects, electrostatic interactions), dynamic sensory, assembly, or phase‐separating properties can be achieved [16, 17]. Additionally, the number of engineered peptides and proteins with non‐native properties is actively expanded, with an on‐going revolution powered by novel computational deep learning tools [18, 19, 20].

As an effect of intensive research toward the development and synthesis of tailored biomolecules, the fields of synthetic and chemical biology have advanced far into the realm of engineering complex biochemical and biological systems [21, 22, 23, 24, 25, 26]. One class of biomolecules that has been extensively studied is coiled‐coil (CC) peptides. These oligomeric peptide assemblies are well‐understood regarding sequence‐structure‐stability relationships [27, 28]. De novo designed CC peptides assemble into α‐helical bundles of two or more peptide strands at adequate conditions, with full control over the oligomeric state, thermal stability and orthogonal assembly of different CC peptide pairs [29, 30, 31]. For example, the CC toolbox contains heterodimeric CCs that exhibit very high selectivity for their respective complementary strands. This leads to a high degree of inertness toward off‐target activities, making these CCs ideal for controlling single functionalities within complex systems. Several heterodimeric CCs have been designed to assemble and disassemble in a fully orthogonal manner, allowing for the use of multiple CC pairs within a single system without cross‐CC interference. A set of such orthogonal CC pairs was designed by Gradišar et al. [31] and applied in biological systems [32]. In addition, CCs have been proven to be excellent vehicles for imparting binding, transportation, or signaling properties into a complex system [29, 33, 34, 35]. As a chemically synthesizable, well‐characterized, and actively expanding class of peptides, they provide excellent prerequisites for use in functional (soft) materials.

**FIGURE 1:**
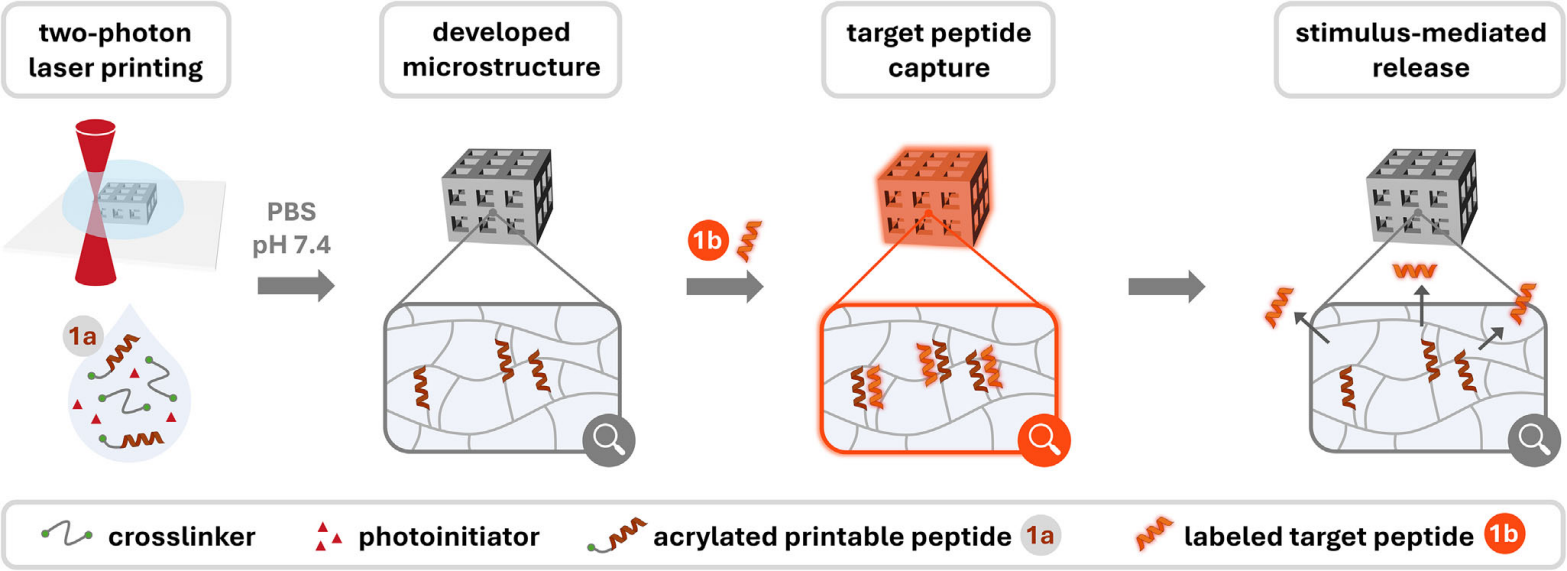
Schematic visualization of fabrication, capture, and release workflow. Acrylamide‐functionalized peptide **1a** is added to a photopolymerizable ink, which is used to print 3D microstructures via 2PLP. After development in phosphate‐buffered saline (PBS), the resultant hydrogel structures can be functionalized with fluorescence‐labeled target peptide **1b** via CC assembly of **1a** and **1b**. Release of the target peptide is mediated by suitable triggers (e.g., temperature or pH changes), which lead to disruption of the CC assembly.

In this work, we present a method for 3D printing of hydrogel microstructures that can selectively bind and release peptides based on specific CC interactions. These structures are fabricated using 2PLP with CC monomers (‘**a**’) as the functional component of the ink. These functionalized microstructures can specifically capture the corresponding CC peptide (‘**b**’) via the peptide handle **a**, forming defined heterodimeric CC structures within the material. To prove our design hypothesis, we use peptides **b** that have been fluorescently labelled to enable characterization of the capture‐and‐release process by the peptide‐functionalized material using fluorescence microscopy. We demonstrate that the captured peptides can be released in response to various stimuli, such as changes in temperature, pH and ionic strength—parameters that disrupt CC interactions (Figure 1). By using sets of orthogonal CC peptide pairs, multimaterial structures can be printed with full spatial resolution over binding of different target peptides in the materials. The target peptides can be orthogonally captured and released, making the system suitable for the production of multifunctional complex hydrogel materials.

## 2 Results and Discussion

### 2.1 Design of Inks for Controlled Peptide Capture and Release

**FIGURE 2:**
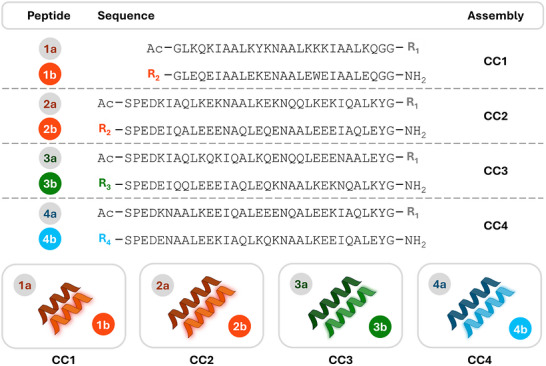
Overview of all synthesized CC‐forming peptides. CC strand ‘**a**’ of each CC pair was acrylamide‐derivatized to be used inside a photopolymerizable ink (R_1_ = tetraethylene‐glycol‐linked acrylamide moiety). Target peptide ‘**b**’ of each CC pair was labeled with a fluorescent dye to be able to microscopically visualize its location. Different fluorescent labels were used to be able to distinguish between the different target peptides within multimaterial structures (R_2_ = tetraethylene‐glycol‐linked 5/6‐carboxy tetramethyl rhodamine (TAMRA) unit, R_3_ = tetraethylene‐glycol‐linked 5/6‐carboxyfluorescein unit, R_4_ = tetra ethylene‐glycol‐linked Cy5.5 unit, Ac = N‐terminal acetyl group, NH_2_ = C‐terminal amide). *Note:* Cy5.5‐labeled peptide **4b** fluoresces in the red color regime but was visualized in cyan color in all figures to increase visual distinction from the TAMRA‐labeled peptide **2b** (orange fluorescence).

Initially, molecular design of the peptide building blocks was performed. To achieve reliable and stable assembly into heterodimeric coiled‐coil structures, we opted for an AB type system, in which A represents a coil strand with a predominantly negative charge and B a coil strand with a predominantly positive charge [36]. The specificity of the assembly of such heterodimeric CCs is mainly driven through electrostatic interactions. Both peptides were synthesized using solid‐phase peptide synthesis. The positively charged peptide, referred to as **1a**, was modified for covalent incorporation into the microstructured material during 2PLP. To this end, a short oligoethylene glycol linker was attached C‐terminally, followed by a lysine residue bearing an acryloyl moiety. The corresponding peptide **1b** was fluorescently labelled to enable characterization of the capture‐and‐release processes in the material by fluorescence microscopy. We chose tetramethyl rhodamine, which was coupled to the N‐terminus of the peptide and was spatially separated from the natural amino acid chain by an oligoethylene glycol linker (Note: An overview of all synthesized peptides can be found in Figure 2). Functionalization at opposite termini of **1a** and **1b** was intentional, to prevent steric hindrance from the crosslinked polymeric network bound to **1a** and the fluorophore label bound to **1b**, while promoting heterodimeric coiled‐coil association in the material.

To be able to print peptide‐containing hydrogels via 2PLP, an optimized ink formulation was developed. First, we tested a composition of peptide‐containing inks that had previously been employed: a mixture of 50 wt.% polyethylene glycol diacrylate (PEGDA, average molecular weight 575 g/mol), 2 wt.% lithium phenyl‐2,4,6‐trimethylbenzoylphosphinate (LAP) and water [10]. During the printing process, the PEGDA component served as a polymerizable, network‐forming crosslinker, while the LAP component acted as a radical photoinitiator. This ink met the main requirements of solubilizing **1a** at elevated concentrations in the mm range while maintaining good printability. However, when we tested the capture of **1b** by soaking the printed structures in phosphate‐buffered saline solutions (PBS, pH 7.4) to form the heterodimeric coiled coil **1a/1b** (**CC1**), we observed that the hydrogels were almost impermeable for **1b**, with diffusion occurring extremely slowly (Figure S26). Consequently, we opted to reduce the degree of crosslinking to yield more permeable hydrogel structures, adding acrylamide (AAm) to the ink formulation as a reactive diluent. Structures printed from these inks exhibited enhanced permeability and favorable diffusion behavior, while retaining excellent printability. The final ink composition for further experiments was set at 34.8 wt.% PEGDA, 31.1 wt.% AAm, 3 wt.% LAP and 31.1 wt.% water. **1a** ink concentration tests showed good solubility at 10 mm. This concentration was used for all subsequent experiments, as concentrations below the mm range decreased the capture ability of the printed structures, while higher concentrations would lead to increased peptide consumption and thus higher synthetic costs. Development of printed microstructures was performed by submersing the samples in water for 1 h followed by submersion in PBS (pH 7.4) for a minimum of 1 h.

**FIGURE 3:**
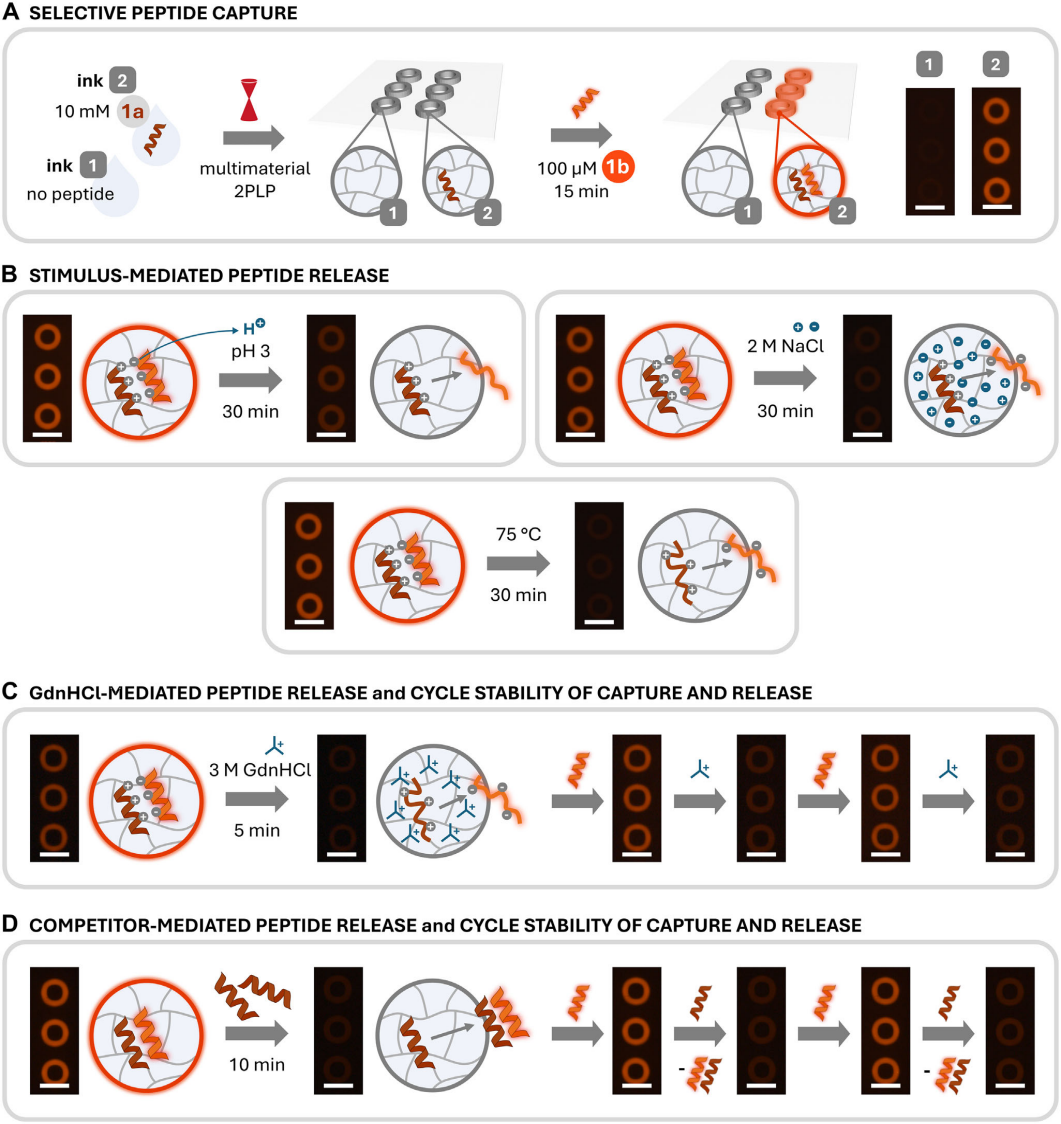
Fluorescence microscopy images of capture and release experiments with 3D‐printed microstructures containing peptide **1a**. (excitation 555 nm; scale bar = 50 µm) (A) Selectivity tests for **1b** peptide capture with multimaterial prints containing structures printed with and without peptide **1a** in the ink. Capture step was performed by submersion of printed structures in a 100 µm **1b** solution in PBS for 15 min. (B) Release studies with different stimuli (pH, ionic strength, temperature) that disrupt the coiled‐coil‐stabilizing ionic and hydrophobic interactions between **1a** and **1b**. (C) Release studies based on the addition of a chaotropic agent (3 m GdnHCl in PBS) onto the printed structures. Capture and release steps are shown to be reversible over three cycles. (D) Selective release studies based on the addition of a solution of competitor peptide **1a** onto the printed structures. **1b** peptides were released from the material by competition over **1b** binding between covalently incorporated **1a** peptides in the material and externally added free **1a** peptide in solution. Capture and release steps are shown to be reversible over three cycles.

After optimizing the ink formulation containing peptide **1a**, the selective capture of **1b** was investigated. First, cylinders with a height of 4–10 µm (50 µm diameter) were printed to examine the diffusion of **1b** inside the printed structures under different capture conditions. The general procedure involved equilibration of the printed and developed structures in a buffered solution of **1b** for a defined duration. After a brief wash with PBS (5‐10 min), the **1b** content within the structures was visualized using fluorescence microscopy. Cylinders measuring 7 µm or less in height exhibited homogeneous fluorescence in the microscopic analysis after a 15 min soaking time in a 100 µm solution of **1b**. However, cylinders with a height of 10 µm height showed increased fluorescence in a corona pattern around the edges. Increasing the soaking time to 30 min or the concentration to 500 µm **1b** marginally improved this effect (Figure S27), indicating limited diffusion of peptide into printed structures due to the bulky geometry. Moving toward microstructures with a higher surface‐to‐volume ratio might improve diffusion [37, 38, 39]. The initial conditions (15 min, 100 µm **1b**) were therefore maintained for subsequent experiments. To ensure fast and complete diffusion with a simple and well visualizable architecture for the systematic analysis of different system parameters, ring‐shaped structures (50 µm diameter, 8 µm lateral ring thickness, 6 µm height) were printed henceforth. To ensure the selective functionalization of the materials through the incorporation of **1a**, as a control, reference structures were printed onto each sample with peptide‐free ink (Figure 3A, Figure S19). Additionally, a labeled version of capture peptide **1a** was synthesized (**1a‐L**). By printing with an ink containing **1a‐L**, homogeneous incorporation of the peptide in the printed structures could be shown via fluorescence microscopy. Incubation in a solution of **1b** demonstrated the functionality of the capture properties. The spatial accordance of **1a‐L** presence and **1b** capture could be demonstrated (Figure S31).

Having optimized the printing conditions for the **1a**‐containing materials and the subsequent capture of **1b**, we proceeded to study the release of **1b** from the microstructures under various conditions. First, the background diffusion‐mediated release of **1b** from the printed microstructures over time was studied (Figure S28B). Therefore, the printed structures were equilibrated in PBS at room temperature. After 1 h, the microstructures exhibited slightly reduced fluorescence (75% of the initial brightness), indicating the loss of some of the captured **1b** peptides. After 24 h, the structures were still visibly fluorescent. However, the brightness had largely decreased to 26% of the initial value, signifying the diffusion of most of the initially captured **1b** peptides into the surrounding buffer. Next, release stimuli that accelerate the diffusion‐mediated **1b** release were investigated (Figure 3B). CCs dissemble into their disordered single strands at acidic pH, high salt concentrations, or elevated temperatures, with the first two conditions disrupting electrostatic interactions. We hypothesized that the release of **1b** should occur under these conditions due to denaturation of the CC structure. Indeed, when the printed structures were equilibrated at pH 3, in 2 m NaCl or at 75°C, almost complete release of **1b** from the microstructures occurred within 30 min.

To further investigate the binding and release of **1b** within the material, two additional release mechanisms promising rapid exchange kinetics were examined: the release of **1b** through the addition of a guanidine hydrochloride (GdnHCl) solution and the release of **1b** through binding competition with non‐immobilized **1a**. GdnHCl is a strong chaotropic salt that is commonly used to denature proteins. Adding a 3 M GdnHCl solution in PBS to the printed structures achieved complete release of **1b** within 5 min. Furthermore, this process could be repeated to capture and release peptide **1b** iteratively within the printed structures (Figure 3C). The addition of a 1 mm solution of free **1a** as an external competitor to the material‐bound **CC1** resulted in the near‐complete release of **1b** from the material within a few minutes, with 10 min determined as a reliable and fast equilibration time period (Figure 3D, Figure S30). Iterative cycles of binding and release of **1b** were also demonstrated in this case. Due to the high selectivity of CC recognition combined with the mild, biocompatible conditions of the binding competition process, this release technique has considerable potential for the development of stimuli‐responsive multimaterial structures in biological environments.

### 2.2 Multimaterial Microstructures for Spatially Resolved Peptide Capture

We envisaged using orthogonal heterodimeric CCs to develop peptide‐functionalized multimaterial structures with spatially resolved peptide capture. Following our initial setup, we synthesized a set of three orthogonal CC pairs, developed by Gradišar et al. [31]. We functionalized each peptide so that one strand of each CC pair was modified with an acrylate moiety for use in 2PLP inks and the other was modified with a fluorescent dye for microscopic localization. The dyes were chosen such that the three orthogonal CC pairs could be visualized separately. The different peptide pairs are referred to as **2a/2b** (**CC2**), **3a/3b** (**CC3**), and **4a/4b** (**CC4**) (Figure 2).

**FIGURE 4:**
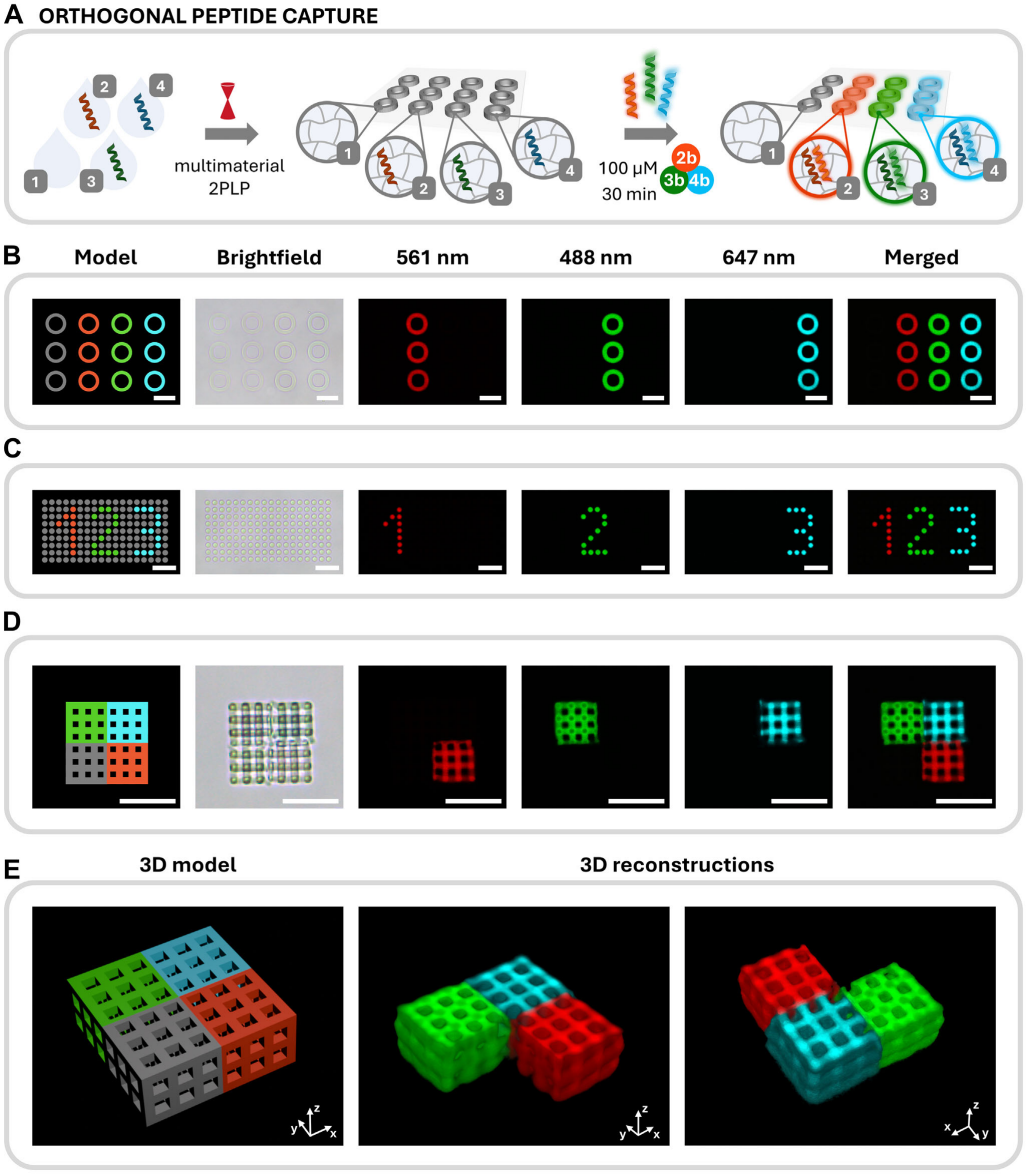
Confocal fluorescence microscopy images of multimaterial microstructures showing spatially resolved peptide capture. (scale bar = 50 µm) (A) Exemplary workflow for orthogonal peptide capture in multimaterial microstructures. Printed and developed structures were submersed in a 100 µm solution of peptides **2b**, **3b**, and **4b** in PBS for 30 min. The labeled peptides were captured with full spatial resolution only in printed areas where the respective complementary peptide **2a**, **3a**, or **4a** was covalently incorporated inside the structures during printing. (B) Ring‐shaped 3D‐printed microstructures displaying spatial capture in the designated columns. (C) 3D‐printed cylinder array exhibiting selective peptide binding, revealing numbers in the respective excitation channels of the confocal microscope that are not visible in brightfield. (D) 3D grid with four different peptide functionalized sectors with either no peptide, peptide **2b**, **3b**, or **4b**. (E) 3D reconstructions of a set of confocal slices at different Z‐levels of the 3D grid.

3D‐printed multimaterial structures were obtained by printing with four different inks containing either no peptide (reference ink), 10 mm **2a**, 10 mm **3a,** or 10 mm **4a**. The inks were sequentially used by performing 2PLP, followed by development in water and subsequent replacement with the next ink. After printing of 3D microstructures with all four inks, a final development step in PBS was performed before further usage. Different architectures were chosen to illustrate the possibilities for creating complex printed systems within a single sample, including rings, number‐coded cylinder arrays, and 3D lattices (Figure 4). Subsequently, a solution containing the three fluorescently labeled complementary strands **2b**, **3b**, and **4b** at 100 µm concentration in PBS was added to the printed and developed structures. The soaking time was set to 30 min to ensure complete diffusion into the printed structures, accounting for the increased chain length of the peptides compared to **CC1** (Figure 4A). After 5–10 min equilibration in PBS, the printed microstructures were visualized using a confocal fluorescence microscope. Successful orthogonal capture with full spatial resolution and no visible off‐target activity within the predefined areas was demonstrated by visualizing the different fluorescent labels within the respective excitation channels of the microscope (561 nm for **2b** visualization, 488 nm for **3b** visualization, 647 nm for **4b** visualization, Figure 4B–D). To visualize the complete diffusion of the labelled peptides into the printed microstructures, we performed a 3D reconstruction of a series of confocal Z‐slice images of the printed 3D grids. As intended, the labelled peptides were present in all the designated areas of the microstructure (Figure 4E).

Building on the high selectivity of orthogonal CC‐directed material functionalization indicated by this study, we investigated whether the respective areas of our multimaterial could be functionalized separately with the same level of selectivity. To this end, we manufactured multimaterial structures with similar functionality and equilibrated them with capture solutions, each of which contained only one of the three target peptides **b** (Figures S34–S36). Indeed, only the material sections functionalized with the complementary coil strand were labelled, and no cross‐reactivity in CC formation was observed. To demonstrate the necessity of CC forming interactions for the capture step, a set of target peptides with scrambled sequences (**2b‐S**, **3b‐S** and **4b‐S**) was synthesized as a negative control. Incubation of multimaterial structures containing areas **2a**, **3a** and **4a** showed no capture of the scrambled target peptides (Figure S33). Finally, we analyzed the potential leaking of coil‐peptides **b** at the interfaces between functionalized and non‐functionalized areas by printing connected “half‐moon” features using reference and peptide‐containing inks. Using fluorescence microscopy, we could not observe any significant undesired peptide diffusion across the material interface (Figure S32).

### 2.3 Orthogonal Peptide Release from Multimaterial Microstructures

Having investigated orthogonal peptide capture in multimaterial structures with spatial and temporal resolution, we evaluated previously developed release methods. While external stimuli such as pH changes and the addition of chaotropic salts promise rapid and complete release of the captured peptides, orthogonal release of the fluorescence‐labelled peptide strands **2b**, **3b** and **4b** from their defined area within the multimaterial structures is desirable to achieve complete spatial and temporal control over the multimaterial system's functions.

**FIGURE 5:**
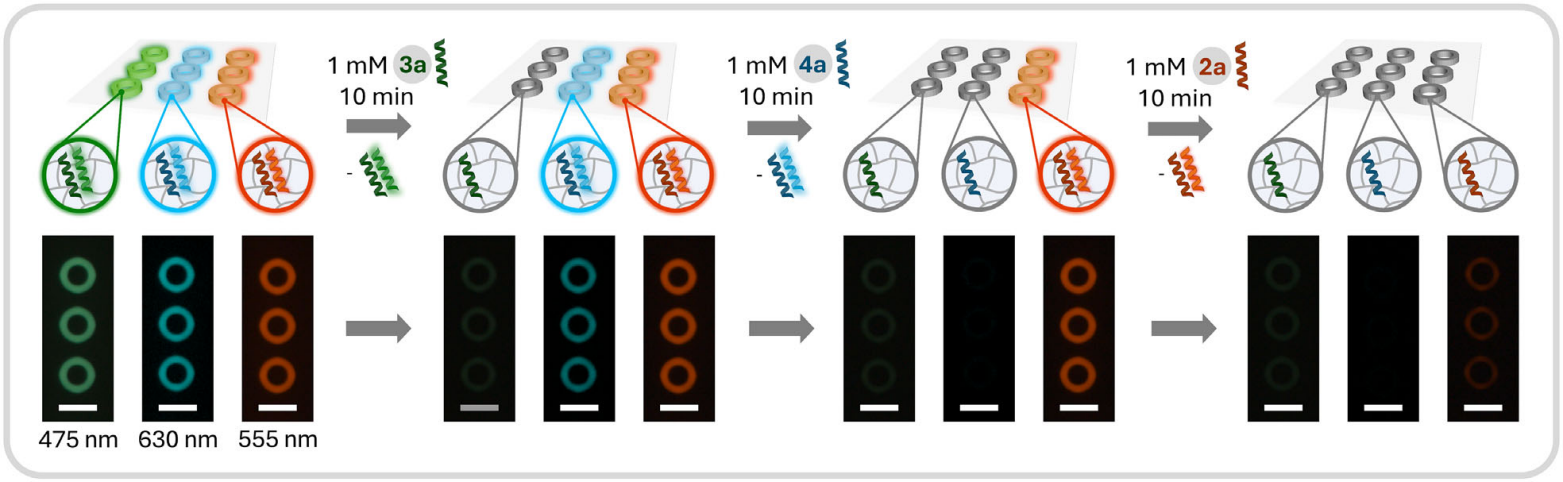
Orthogonal competitor‐mediated peptide release in multimaterial microstructures. (scale bar = 50 µm) Multimaterial print containing areas with captured peptides **2b**, **3b**, and **4b**, respectively. Stepwise release of peptides **3b**, **4b**, and **2b** in this order was achieved by subsequent submersion of the printed structures in 1 mm competitor solutions.

We then focused on orthogonal release mediated by a competitor, as we anticipated that this would provide a high level of selectivity during the release process under mild conditions. In order to determine the most sensible order of peptide release, background diffusion of the labelled peptide strands from the printed structures was tested. Tracking the loss of fluorescence over 1 h showed that peptide **3b** was released fastest, followed by **4b** (Figure S37). Hence, orthogonal competitor‐mediated release was performed in the order **CC3**→**CC4**→**CC2**. We tested different release conditions, starting with the previously optimized parameters (1 mm competitor in PBS, 10 min, Figure 5). Under these conditions, we observed near‐complete release of **3b**, while **4b** and **2b** remained bound to their defined areas within the material. When an excess of unbound **4a** was added as a competitor, only **4b** was released while **2b** remained in the material. However, slow background release of **2b** and **4b** during the initial release of **3b** was observed, resulting in a slight loss of fluorescence in the material sections functionalized with **CC2** and **CC4**. This effect was particularly evident in the case of **CC4**. These findings were in line with the background diffusion tests, which showed significant diffusion from the printed structures after 1 h of equilibrating the samples in PBS. To accelerate the release kinetics, we increased the concentration of the competitors to 10 mm (Figure S39A). This resulted in near‐identical fluorescence brightness for **CC2** and **CC4** after selective release of **CC3** compared to our initial conditions with the lower competitor concentration of 1 mm. It also indicates that the unwanted reduction in fluorescence of **CC2** and **CC4** is due to background diffusion rather than undesired CC cross‐interactions. A similar result was achieved when adding 1 m GdnHCl to promote the release process at 1 mm competitor concentration (Figure S39B). Additional tests involving varying release times, competitor concentrations, and a different order of release (Figure S39) produced less satisfactory results without decreasing the effect of minor background diffusion. On top of competitor‐mediated procedures, selective release via temperature control was tested. CC pairs **CC2**, **CC3** and **CC4** display melting temperatures that differ by at least 20 K (∼85°C for **CC2**, ∼65°C for **CC3**, and ∼45°C for **CC4**) [31]. However, these distinctive properties in solution were not observed in printed structures. Release tests at 55°C showed unsatisfactory selectivity for the subsequent release of **2b**, **3b** and **4b** (Figure S38).

## 3 Conclusion

In summary, we have shown that discretely folded, chemically synthesized peptides can be used as functional units for 2PLP‐manufactured microstructures. Printing formulation containing acrylamide‐derivatized coil peptides **a,** which can form heterodimeric CC assemblies with complementary coil peptides **b**, was successfully used for the generation of 3D microstructures. The resulting microstructures were found to selectively and efficiently capture peptides **b** through CC interaction, with no nonspecific binding of **b** being observed on the material surface. When exposed to certain environmental conditions such as changes in temperature or pH, peptides **b** were observed to be released rapidly from the material. We also demonstrated that the capture and release mechanism is replicable over multiple cycles for both GdnHCl‐ and competition‐mediated release strategies. By integrating a set of three orthogonal CC peptide pairs into our material design, we succeeded in producing multimaterial structures that enable spatial and temporal control over peptide capture and release. Multimaterial 3D microstructures with defined areas for the selective binding of different target peptides (**2b**, **3b**, and **4b**) were functionalized in a site‐selective manner by simply adding a mixture of these three peptides. This spatiotemporal control over the system was also demonstrated for the subsequent release of the target peptides. Equilibrating the functionalized microstructures in a solution of competitors to the covalently incorporated peptides **a**, peptides **b** could be released through selective CC‐interaction‐mediated exchange.

To our knowledge, the manufacturing system presented herein is the first to incorporate discretely folded synthetic peptides into 2PLP‐manufactured materials. Previous studies utilizing proteins or peptides in this context have focused either on basic sequence‐derived functions (e.g. cell‐adhesive RGD peptides), or on their use as network‐defining structural components (e.g. collagen‐based materials) [9, 10, 11, 12, 13, 14, 15]. By using discretely folded CC peptides as functional components, we were able to transfer their selective assembly properties observed in solution to 3D‐printed materials. This report demonstrates the enormous potential of stimulus‐induced capture and release in 3D‐printed microstructures mediated by assembly and disassembly of CCs. Rather than functionalizing the coil peptides with fluorescent labels, other modifications or cargos such as peptides, proteins, carbohydrates, nucleic acids or small molecules can be chemically attached either during the peptide synthesis process or via a late‐stage functionalization approach (e.g. by click chemistry) [40, 41, 42, 43, 44]. Spatiotemporal control over the capture and release of such compounds in and from 3D‐printed materials is of particular interest for applications in complex biochemical and biological systems. Both the capture and release steps can be performed under physiological conditions and can therefore be considered fully bioorthogonal. Additionally, applications of the platform as a responsive, multifunctional microsystem can be envisaged. For example, reversible loading with catalytically active units could be used for microreactor design, while recyclable sensing systems could be created by deliberately capturing and releasing analyte‐binding units. Affinity‐based separation platforms could also be developed via the efficient capture‐and‐release mechanism of CC‐labeled target molecules.

The system we have presented here demonstrates only a small fraction of the functionalization possibilities arising from the combination of de novo design of CCs and 3D microprinting. The extensive toolbox of CC peptides, each with different properties, holds great potential for further refinement of the capture‐and‐release system. For example, peptide exchange could be promoted by hierarchically ordered systems comprising peptides of different chain lengths [45, 46]. Additionally, CC pairs that respond differently to stimuli (e.g., pH changes) could be investigated to develop materials that enable peptide capture and release with orthogonal stimuli [45, 47, 48, 49]. Further applications beyond the mere reversible functionalization of materials through CC dimerization are conceivable. For example, CCs with receptor and catalytic functions, as well as switchable CCs, have been reported – all of which would add an extra functional dimension to potential 3D‐printed materials [34, 50, 51].

## Conflicts of Interest

The authors declare no conflicts of interest.

## Supporting information




**Supporting File**: advs76505‐sup‐0001‐SuppMat.pdf.

## Data Availability

Additional data are available in the Supplementary Information. The full raw data set is published in the public data repository heiDATA of Heidelberg University (doi.org/10.11588/DATA/FUKIOR).
