# Supplementary material for: Controlled Peptide Capture and Release in 3D‐Printed Multimaterial Microstructures
Source: Adv Sci (Weinh). 2026 Jul 16:e76505. Online ahead of print. doi: 10.1002/advs.76505 (PMC13373895; doi:10.1002/advs.76505)
Supplement: Supplementary file 1 — Supporting File: advs76505‐sup‐0001‐SuppMat.pdf. [file ADVS-9999-e76505-s001.pdf]

Controlled Peptide Capture and Release in 3D-Printed Multimaterial Microstructures

Niklas Schwegler, Thomas Heim, Philipp Mainik, Eva Blasco, and Franziska Thomas**

Table of Contents

1 Data analysis and visualization S2

2 Reagents and solvents S2

3 Experimental procedures S2

4 Peptide modifications S5

5 Peptide characterization S6

6 Fluorescence microscopy S18

1 Data analysis and visualization

Chemical syntheses and structures, including molecular properties, were visualized and analyzed with *ChemDraw 22.2*. HPLC and MALDI-TOF MS data were analyzed and visualized with *OriginPro 2021*. Microscopy images were processed with *ImageJ 1.54p*. Figure arrangement and visualizing schemes were generated with *Microsoft Office 365 PowerPoint* and *Inkscape 1.2*.

2 Reagents and solvents

All reagents and solvents were used as received from the respective suppliers. Ultrapure water was derived from deionized tap water using a *Sartorius* arium® mini water purification system. Phosphate-buffered saline (PBS) was prepared using ultrapure water, potassium dihydrogen phosphate (1.8 mM), disodium hydrogen phosphate (10 mM), sodium chloride (137 mM), and potassium chloride (2.7 mM) and adjusted to pH 7.4.

5-/6-Carboxytetramethylrhodamine, Cy5.5 acid, Fmoc-NH-PEG(4)-OH (protected tetraethylene glycol linker), and Lithium phenyl-2,4,6-trimethylbenzoylphosphinate (LAP) were ordered from *BLD Pharmatech GmbH* (Reinbek, Germany). Dimethylformamide (DMF, peptide synthesis grade) was obtained from *Fisher Scientific GmbH* (Schwerte, Germany). Fmoc-protected standard amino acids, N,N-diisopropylcarbodiimide (DIC), Fmoc-Lys(Acryloyl)-OH, and Tentagel XV Rink Amide resin (0.24 mmol/g) were purchased from *Iris Biotech GmbH* (Marktredwitz, Germany). Acetonitrile (MeCN, HPLC grade), acrylamide (AAm), 5(6)-carboxyfluorescein (FAM), N,N-diisopropylamine (DIPEA), Oxyma® Pure (Novabiochem®), piperidine, polyethylene glycol diacrylate (PEGDA, MW 575), PyBOP (Novabiochem®), trifluoroacetic acid (TFA, HPLC grade), and triisopropylsilane (TIPS) were obtained from *Merck KGaA* (Darmstadt, Germany).

All listed and unlisted chemicals were purchased in pro-analytical or synthesis grade. Unlisted chemicals were obtained from *BLD Pharmatech GmbH* (Reinbek, Germany), *Carl Roth GmbH + Co. KG* (Karlsruhe, Germany), *Fisher Scientific GmbH* (Schwerte, Germany), and *Merck KGaA* (Darmstadt, Germany).

3 Experimental procedures

Solid-phase peptide synthesis

Automated SPPS was performed using 0.2 M Fmoc-protected amino acid solutions, a 0.5 M DIC solution (activator), a 1.0 M Oxyma® solution containing 0.1 M DIPEA (activator base), and a 20 vol% piperidine solution (deprotection) in DMF. Synthesis was carried out with a CEM Liberty Blue device using standard single and double coupling procedures. 200 mg of Tentagel XV Rink Amide resin (0.24 mmol/g) were used per peptide (48 µmol scale). Following synthesis, the resin was transferred to a syringe equipped with a PE frit, washed sequentially with 5×5 mL DMF, 5 mL dichloromethane, 5 mL methanol, and again 5 mL dichloromethane, and subsequently dried under vacuum for further processing. Manual SPPS was performed for N-terminal dye functionalization of peptides **1b**, **1a-L**, **2b**, **3b**, **4b**, **2b-S**, **3b-S**, and **4b-S**. A mixture of 2 eq. dye acid, 2 eq. PyBOP, and 4 eq. DIPEA in DMF with respect to the peptide amount on solid phase were added to the resin in a syringe equipped with a PE frit and equilibrated on a shaker for 2 h. Resin was then washed sequentially with 5×5 mL DMF, 5 mL dichloromethane, 5 mL methanol, and again 5 mL dichloromethane, and subsequently dried under vacuum for further processing.

N-terminal acetylation of peptides **1a**, **2a**, **3a**, and **4a** was performed by adding a mixture of 4.5 mL pyridine and 0.5 mL acetic anhydride to the resin and equilibrating on a shaker for 15 min. Resin was then washed sequentially with 5×5 mL DMF, 5 mL dichloromethane, 5 mL methanol, and again 5 mL dichloromethane, and subsequently dried under vacuum for further processing. Peptide cleavage from the resin was carried out by adding a mixture of 0.25 mL water, 0.25 mL TIPS, and 4.5 mL TFA to the resin-containing syringe and equilibrating on a shaker for 3 h. The resultant solution was extracted from the resin, including washing of the resin with 2×3 mL TFA. Volume of the combined liquids was reduced to ~2 mL by evaporation of TFA with a continuous nitrogen stream. The crude peptide was precipitated with ~20 mL ice-cold diethyl ether and extracted by centrifugation, including two additional washes with ~20 mL ice-cold diethyl ether. Crude peptide precipitate was dried under vacuum and dissolved in a water/MeCN mixture containing 0.1% TFA for purification.

High-performance liquid chromatography

Peptides were purified by reverse-phase high-performance liquid chromatography (HPLC) under yellow light conditions on a Shimadzu system equipped with a CBM-40 system controller, a DGU-405 degassing unit, a LC-40D solvent delivery module, a SPD-M40 photo diode array detector and a CTO-40S column oven using a VDS optilab VDSpher® PUR 100 C18-SE column (250 mm × 10 mm, 100 Å, 5 µm). The flow rate was set to 3 mL min^{-1} and the oven temperature maintained at 50 °C. Analytical HPLC was performed on a Hitachi system with a 1110 pump, 1210 autosampler, 1310 column oven, and 1430 diode array detector, using a VDSpher® PUR 100 C18-SE column (250×4.6 mm, 100 Å, 5 µm; VDS optilab Chromatographie-technik GmbH) at a flow rate of 1 mL min^{-1} and 50 °C oven temperature. Chromatograms were recorded at 220 nm and 280 nm. The solvent system consisted of buffer A (water with 0.1% TFA) and buffer B (MeCN with 0.1% TFA). Gradient programs "X-Y% B in A" were designed in the following manner: 5 min equilibration at X% B in A was followed by a 30 min gradient from X% B in A to Y% B in A. Subsequent 15 min were programmed for washing the column at 95% B in A and re-equilibration to X% B in A for the next run (total time per run: 50 min). The respective utilized gradient programs are indicated separately for each chromatogram.

Mass spectrometry

Matrix-assisted laser desorption ionization time-of-flight mass spectrometry (MALDI-TOF MS) was performed on an Autoflex Speed spectrometer (Bruker Corporation). Samples were spotted onto a steel target plate by mixing 0.8 µL of sample solution with 0.8 µL of matrix solution (20 mg mL^{-1} 2,5-dihydroxybenzoic acid in 30% MeCN/0.1% TFA/water), and subsequent air-drying. The employed measurement parameters are separately indicated for each spectrum.

Silanization procedure

Full procedure was performed under yellow light conditions. Glass substrates (22×22 mm, thickness 170 ± 5 µm; Paul Marienfeld GmbH & Co. KG) were cleaned with isopropanol and acetone, dried under nitrogen, and plasma-activated in a Harrick plasma cleaner (high mode, 40-50 min). Substrates were treated with a 4 mM solution of 3-(trimethoxysilyl)propyl acrylate in toluene over night. Before usage, substrates were rinsed with toluene (2×) and acetone and dried under nitrogen.

Ink preparation

Full procedure was performed under yellow light conditions. LAP was dissolved in a mixture of water, PEGDA (MW 575), and AAm (ratio 1:1:1/V:V:m) to a final composition of 3.0 wt% LAP (reference ink). For peptide containing inks, respective volumes of reference ink solution were added to aliquots of lyophilized pure peptide, yielding an ink solution of the desired 10 mM peptide concentration.

Two-photon laser printing

Microstructures were fabricated using a Photonic Professional GT2 system (Nanoscribe GmbH) with a 780 nm femtosecond laser focused through a 25× NA0.8 oil immersion objective (Carl Zeiss Microscopy GmbH). STL files were generated with Blender 4.3 and processed using the DeScribe software package (Nanoscribe GmbH) with slicing and hatching distances set to 0.3 µm, applying a 90° hatching angle offset per slice and antiparallel hatching. Structures were written at 40% laser power (100% corresponding to 50 mW per calibration) with a scan speed of 4 mm s^{-1} for 3D grids and 5 mm s^{-1} for all other structures. Inks were applied on silanized glass substrates using a PDMS mold sealed with a glass coverslip to prevent evaporation. For multimaterial printing, structures were immersed in water for 5 min, and the PDMS mold was rinsed with the new ink by pipetting up and down 3×10 times before refilling with fresh ink and resealing. Printed structures were developed in water for 1 h and washed in 1× PBS (pH 7.4) for a minimum of 1 h before further usage. Samples were stored in 1× PBS at 4 °C and further experiments were performed within 24 h.

Fluorescence microscopy

Fluorescence images were recorded using a Zeiss Axio Imager.Z2m equipped with a Zeiss Epiplan-NEOFLUAR 10× NA0.25 objective. Used excitation wavelengths (475 nm, 555 nm, 630 nm) are indicated for each respective displayed image. True color images were used for 475 nm and 555 nm excitation. For visualization purposes (good color distinction between 555 nm and 630 nm images), 630 nm images were transferred to grayscale followed by application of a cyan color filter.

Confocal fluorescence microscopy

Confocal fluorescence images were recorded using a Nikon A1R confocal microscope equipped with GaAsP-detectors. For 2D confocal images, a Nikon 20× NA0.8 objective was used with the pinhole set to 1.0 AU. For z-stacks of printed grids for 3D reconstruction, a Nikon 60× NA1.4 oil immersion objective was used at pinhole 1.0 AU and a step size of 0.1 µm with 211 total slices per stack.

4 Peptide modifications

Chemical structures of the non-natural amino acids and modifications of the presented peptides are visualized in the following. Occurrence of the chemical fragments shown with respect to the synthesized peptides are indicated in the figure.

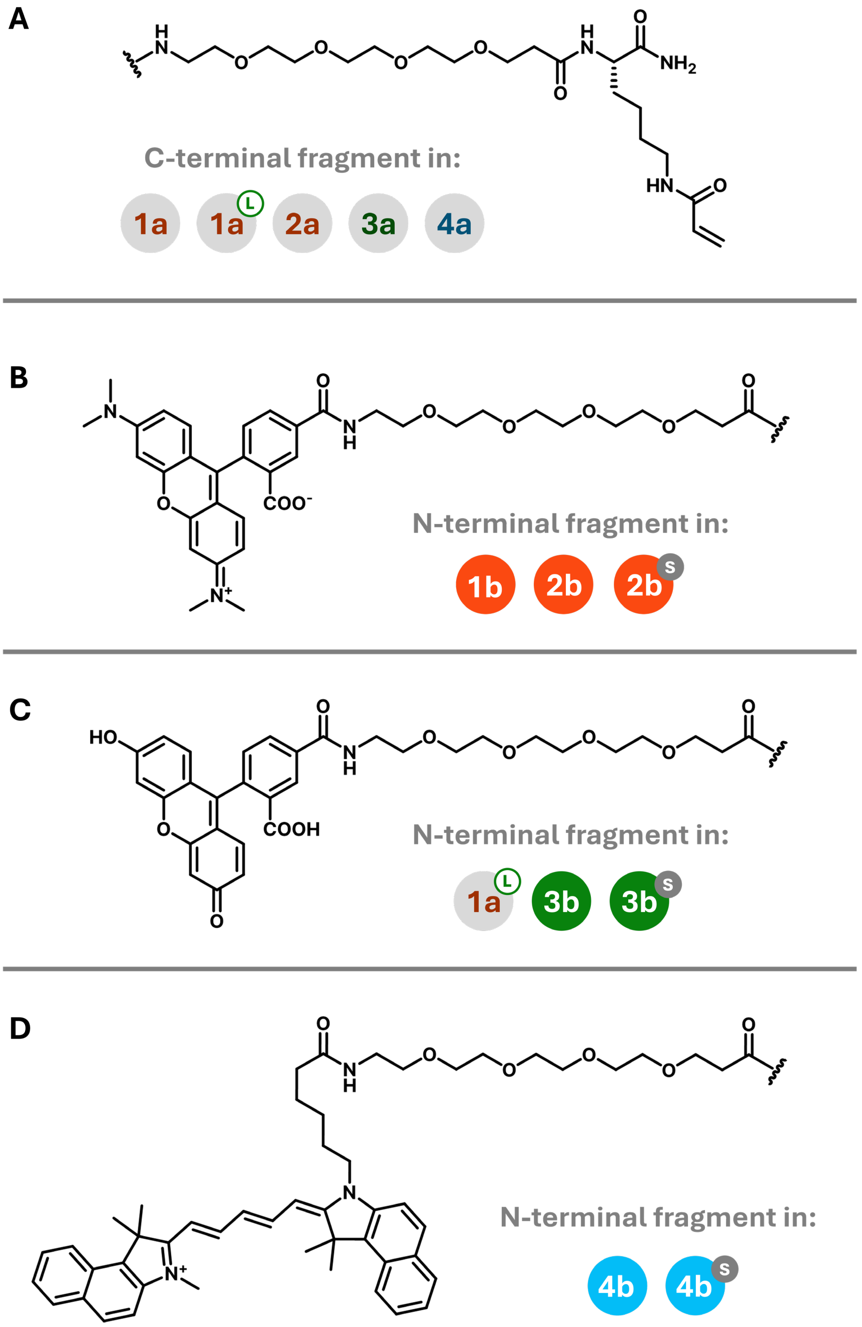

Figure S 1: *Chemical structure of N- and C-terminal modifications in the presented peptides.* ***(A)*** *C-terminal fragment containing a tetra ethylene glycol linker and a modified lysine unit bearing an acrylamide moiety. Contained in peptides* ***1a, 1a-L, 2a, 3a, 4a.*** ***(B)*** *N-terminal fragment containing a tetra ethylene glycol linker and a 5-TAMRA dye unit. Contained in peptides* ***1b, 2b, 2b-S****. Note: An isomeric mixture of 5-/6-TAMRA was used for synthesis.* ***(C)*** *N-terminal fragment containing a tetra ethylene glycol linker and a 5-FAM dye unit. Contained in peptides* ***1a-L, 3b*** *and* ***3b-S****. Note: An isomeric mixture of 5-/6-TAMRA was used for synthesis.* ***(D)*** *N-terminal fragment containing a tetra ethylene glycol linker and a Cy-5.5 dye unit. Contained in peptides* ***4b*** *and* ***4b-S****.*

5 Peptide characterization

Peptide 1a

Ac-GLKQKIAALKYKNAALKKKIAALKQGG-X-Z-NH_2

(Ac = acetyl, X = tetraethylene glycol linker (CAS 557756-85-1), Z = N-ε-acryloyl-L-lysyl)

$C_{153}H_{272}N_{42}O_{39}$

Monoisotopic mass: 3322.1 Da

Molecular weight: 3324.1 g mol^{-1}

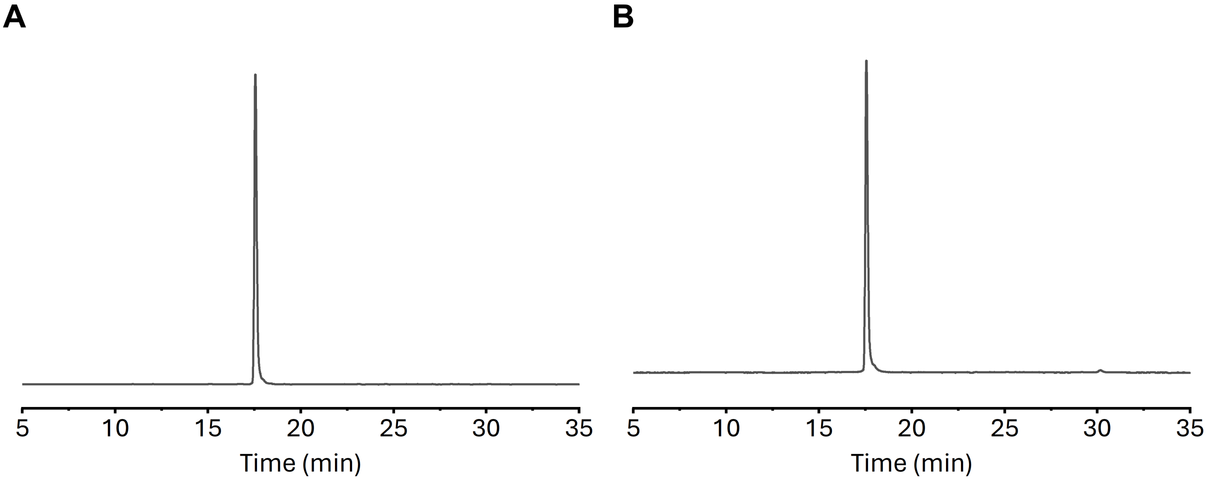

Figure S 2: *Analytical HPLC traces of pure **1a** (20-50% A in B). **(A)** Chromatogram at 220 nm. **(B)** Chromatogram at 280 nm.*

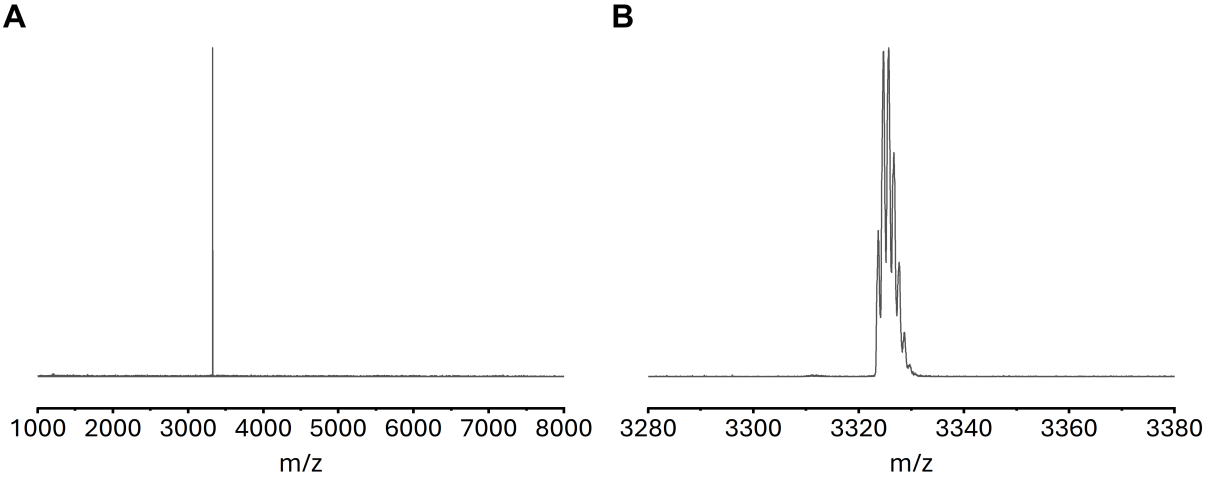

Figure S 3: *MALDI-TOF MS spectra of **1a** (RP1000-8000 mode). **(A)** Full spectrum. **(B)** Zoom in on product isotope pattern. <u>Peaks:</u> $[M+H]^+$ calc. 3323.1 m/z, found 3323.7 m/z.*

Peptide 1a-L

Z_1-X-GLKQKIAALKYKNAALKKKIAALKQGG-X-Z_2-NH_2

(X = tetraethylene glycol linker (CAS 557756-85-1), Z_1 = 5-/6-carboxyfluorescein-yl, Z_2 = N-ε-acryloyl-L-lysyl)

$C_{183}H_{301}N_{43}O_{49}$

Monoisotopic mass: 3885.2 Da

Molecular weight: 3887.7 g mol^{-1}

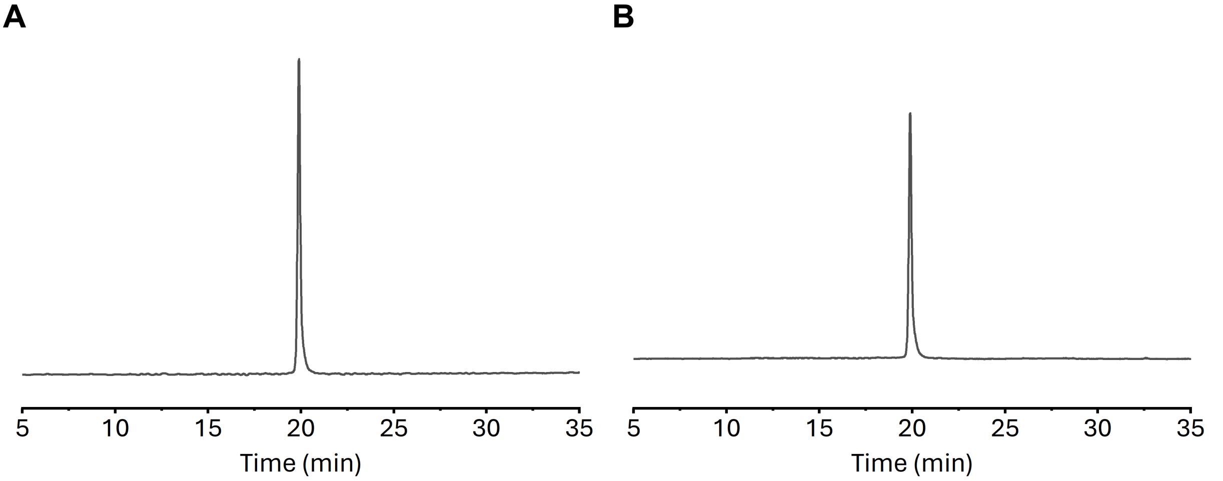

Figure S 4: *Analytical HPLC traces of pure **1a-L** (20-50% A in B). **(A)** Chromatogram at 220 nm. **(B)** Chromatogram at 280 nm.*

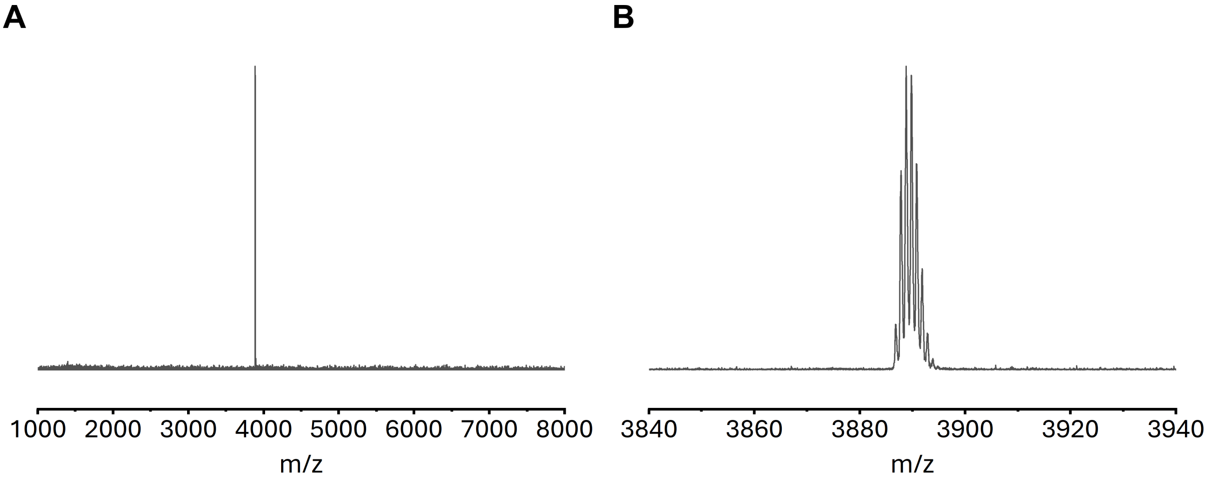

Figure S 5: *MALDI-TOF MS spectra of **1a-L** (RP1000-8000 mode). **(A)** Full spectrum. **(B)** Zoom in on product isotope pattern. <u>Peaks:</u> $[M+H]^+$ calc. 3886.2 m/z, found 3886.8 m/z.*

Peptide 1b

Z-X-GLEQEIAALEKENAALEWEIAALEQGG-NH_2

(X = tetraethylene glycol linker (CAS 557756-85-1), Z = 5-/6-carboxytetramethylrhodamine-yl)

$C_{162}H_{242}N_{36}O_{53}$

Monoisotopic mass: 3540.7 Da

Molecular weight: 3541.9 g mol^{-1}

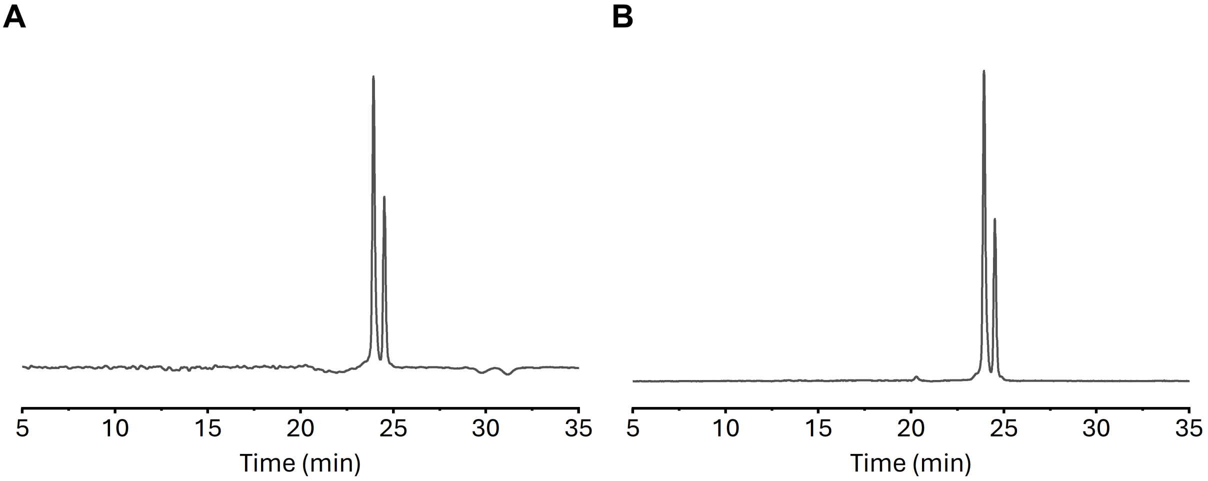

Figure S 6: *Analytical HPLC traces of pure* ***1b*** *(30-60% A in B).* *Note:* *The presence of two peaks is attributed to the use of an isomeric mixture of 5-/6-TAMRA for labeling. The two isomers could not be separated via HPLC for* ***1b****, hence an isomeric mixture was used.* ***(A)*** *Chromatogram at 220 nm.* ***(B)*** *Chromatogram at 280 nm.*

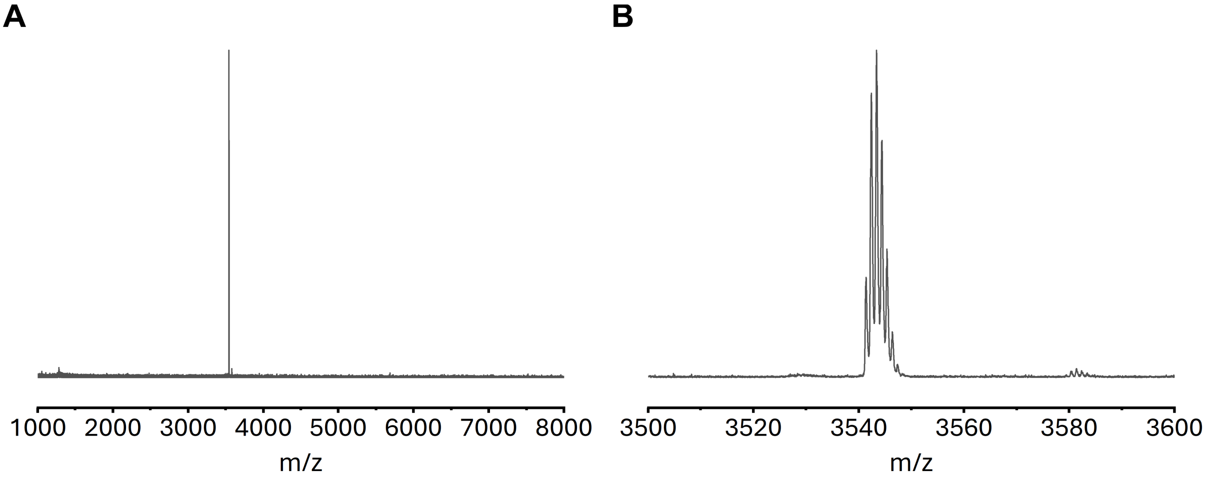

Figure S 7: *MALDI-TOF MS spectra of* ***1b*** *(RP1000-8000 mode).* ***(A)*** *Full spectrum.* ***(B)*** *Zoom in on product isotope pattern.* *Peaks:* *$[M+H]^+$ calc. 3540.7 m/z, found 3541.4 m/z.*

Peptide 2a

Ac-SPEDKIAQLKEKNAALKEKNQQLKEKIQALKYG-X-Z-NH_2

(Ac = acetyl, X = tetraethylene glycol linker (CAS 557756-85-1), Z = N-ε-acryloyl-L-lysyl)

$C_{189}H_{323}N_{51}O_{59}$

Monoisotopic mass: 4251.4 Da

Molecular weight: 4254.0 g mol^{-1}

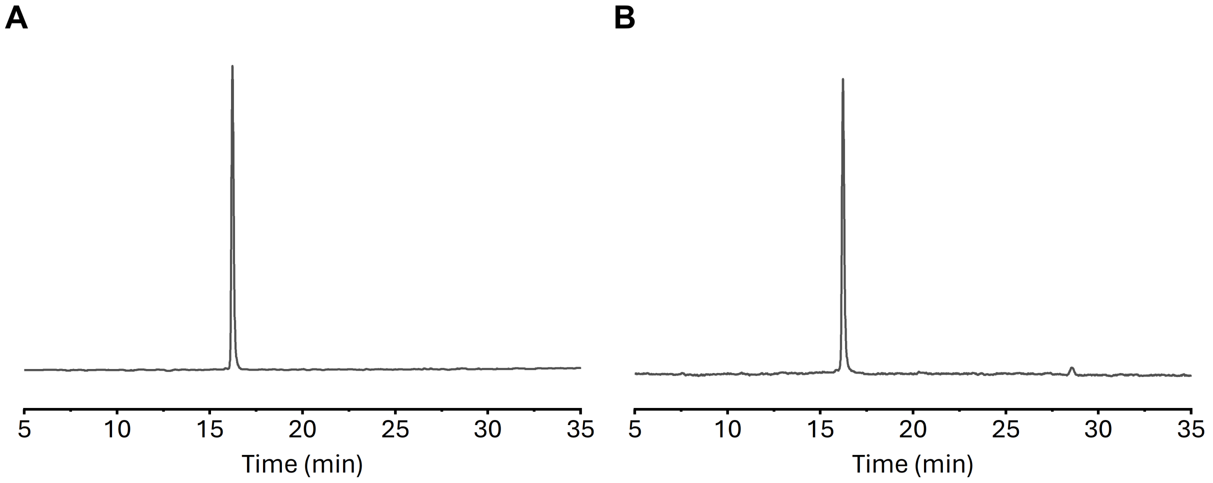

Figure S 8: *Analytical HPLC traces of pure* ***2a*** *(20-50% A in B).* ***(A)*** *Chromatogram at 220 nm.* ***(B)*** *Chromatogram at 280 nm.*

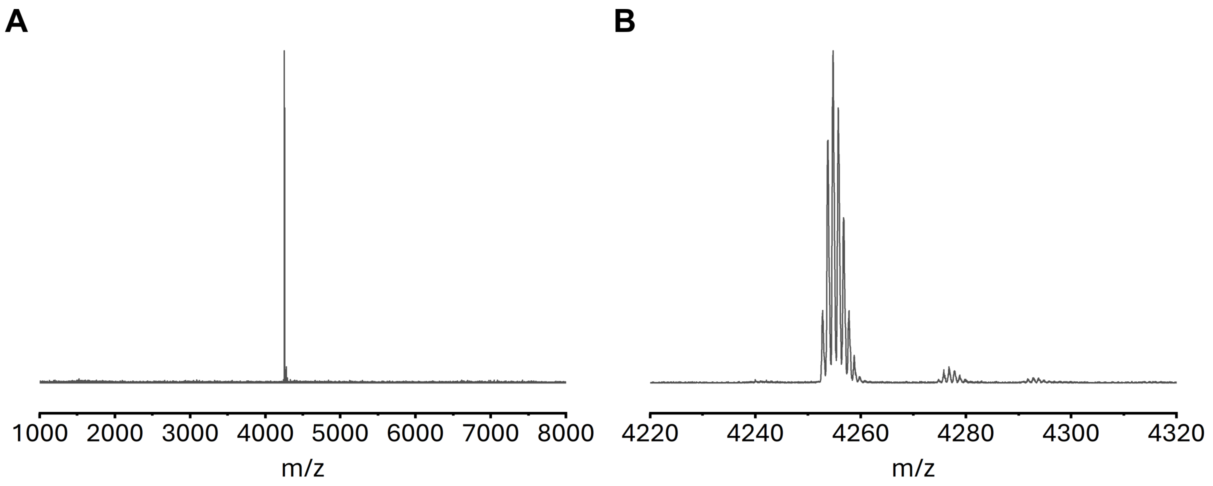

Figure S 9: *MALDI-TOF MS spectra of* ***2a*** *(RP1000-8000 mode).* ***(A)*** *Full spectrum.* ***(B)*** *Zoom in on product isotope pattern. Peaks: $[M+H]^+$ calc. 4252.4 m/z, found 4252.8 m/z; $[M+Na]^+$ calc. 4274.4 m/z, found 4274.8 m/z.*

Peptide 2b

Z-X-SPEDEIQALEEENAQLEQENAALEEEIAQLEYG-NH_2

(X = tetraethylene glycol linker (CAS 557756-85-1), Z = 5-/6-carboxytetramethylrhodamine-yl)

$C_{193}H_{285}N_{43}O_{74}$

Monoisotopic mass: 4389.0 Da

Molecular weight: 4391.6 g mol^{-1}

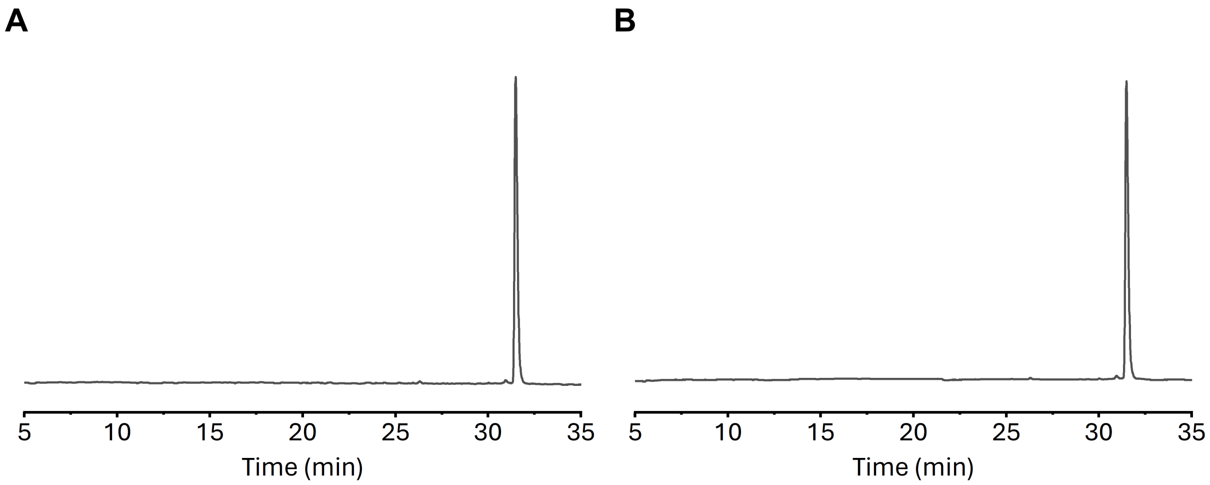

Figure S 10: *Analytical HPLC traces of pure* ***2b*** *(20-50% A in B).* ***(A)*** *Chromatogram at 220 nm.* ***(B)*** *Chromatogram at 280 nm.*

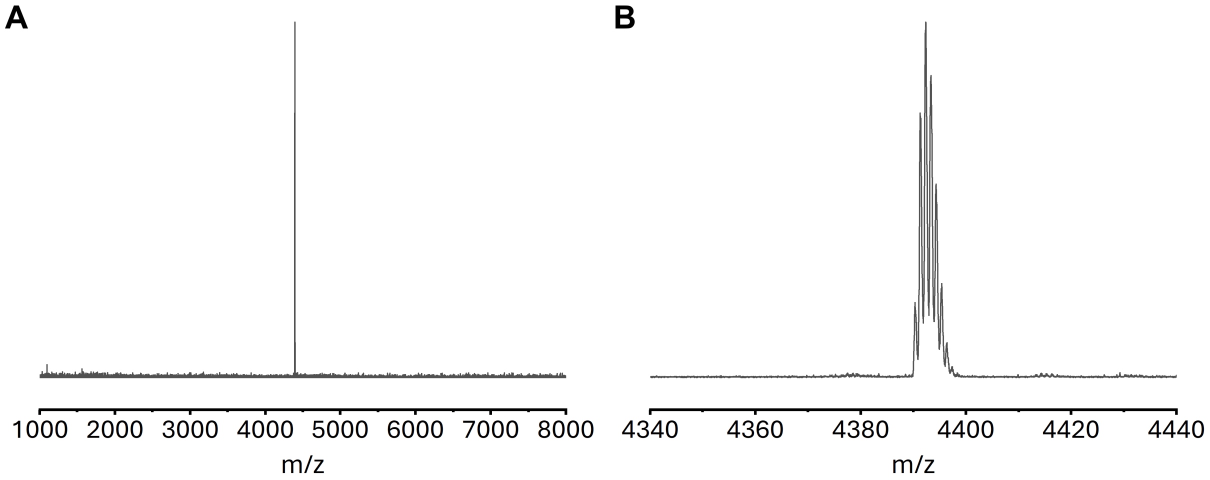

Figure S 11: *MALDI-TOF MS spectra of* ***2b*** *(RP1000-8000 mode).* ***(A)*** *Full spectrum.* ***(B)*** *Zoom in on product isotope pattern. <u>Peaks:</u> $[M+H]^+$ calc. 4390.0 m/z, found 4390.3 m/z.*

Peptide 3a

Ac-SPEDKIAQLKQKIQALKQENQQLEEENAALEYG-X-Z-NH_2

(Ac = acetyl, X = tetraethylene glycol linker (CAS 557756-85-1), Z = N-ε-acryloyl-L-lysyl)

$C_{185}H_{305}N_{49}O_{65}$

Monoisotopic mass: 4253.2 Da

Molecular weight: 4255.8 g mol^{-1}

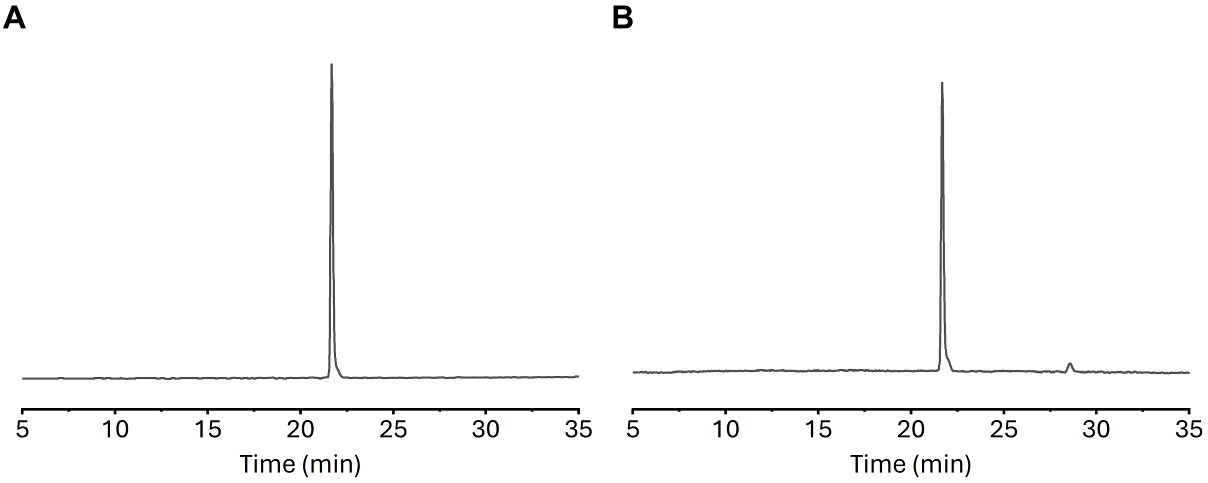

Figure S 12: *Analytical HPLC traces of pure* ***3a*** *(20-50% A in B).* ***(A)*** *Chromatogram at 220 nm.* ***(B)*** *Chromatogram at 280 nm.*

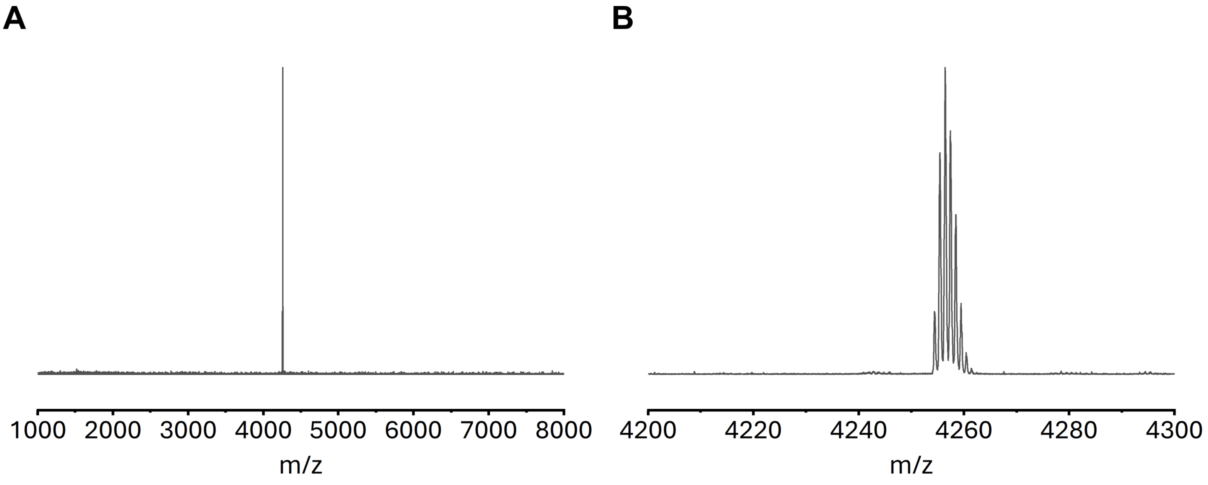

Figure S 13: *MALDI-TOF MS spectra of* ***3a*** *(RP1000-8000 mode).* ***(A)*** *Full spectrum.* ***(B)*** *Zoom in on product isotope pattern. <u>Peaks:</u> $[M+H]^+$ calc. 4253.2 m/z, found 4253.4 m/z.*

Peptide 3b

Z-X-SPEDEIQQLEEEIAQLEQKNAALKEKNQALKYG-NH_2

(X = tetraethylene glycol linker (CAS 557756-85-1), Z = 5-/6-carboxyfluorescein-yl)

$C_{195}H_{298}N_{46}O_{69}$

Monoisotopic mass: 4388.1 Da

Molecular weight: 4390.8 g mol^{-1}

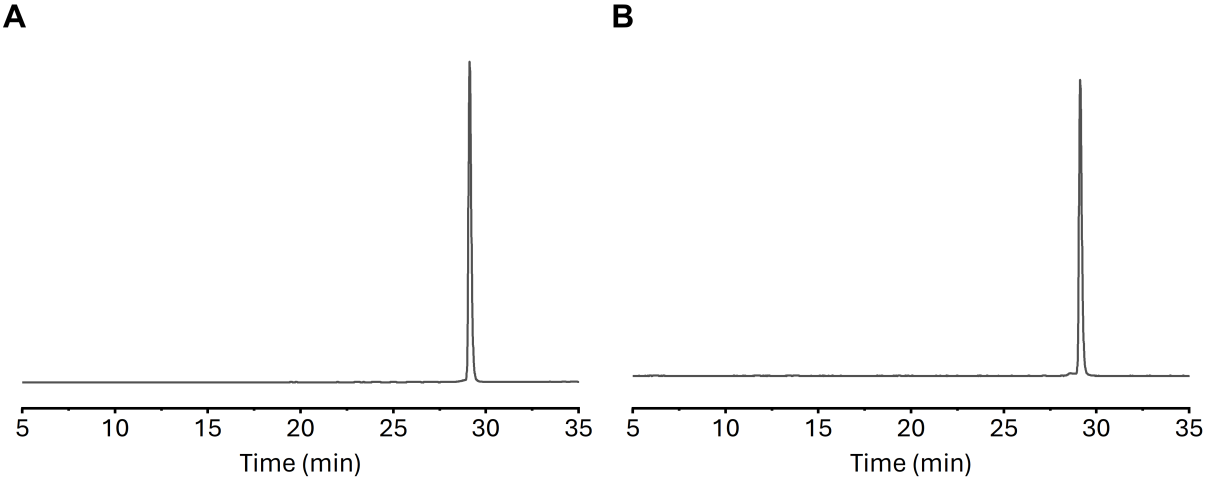

Figure S 14: *Analytical HPLC traces of pure* ***3b*** *(20-50% A in B).* ***(A)*** *Chromatogram at 220 nm.* ***(B)*** *Chromatogram at 280 nm.*

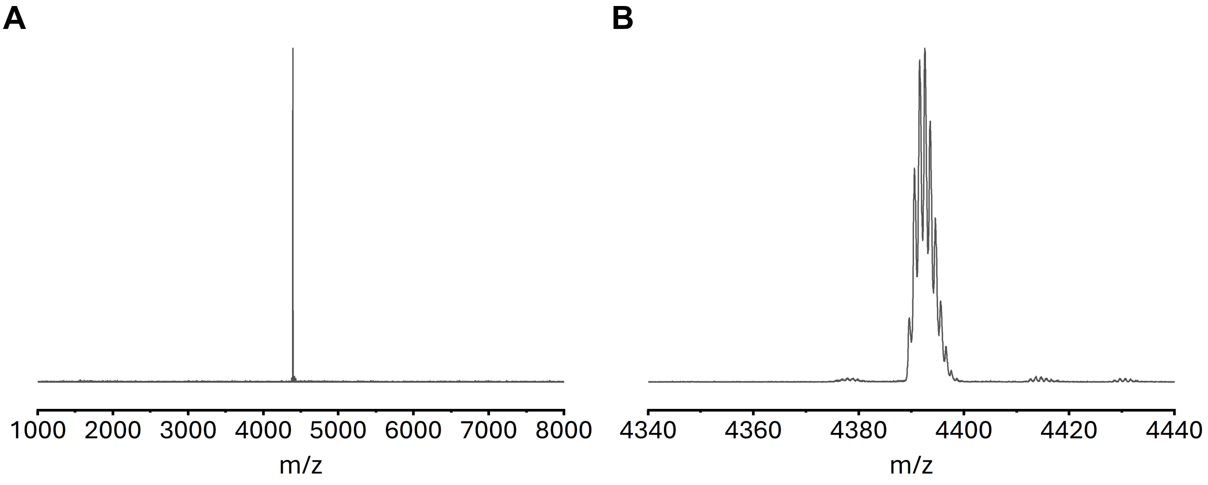

Figure S 15: *MALDI-TOF MS spectra of* ***3b*** *(RP1000-8000 mode).* ***(A)*** *Full spectrum.* ***(B)*** *Zoom in on product isotope pattern.* *<u>Peaks:</u> $[M+H]^+$ calc. 4389.1 m/z, found 4389.6 m/z.*

Peptide 4a

Ac-SPEDKNAALKEEIQALEEENQALEEKIAQLKYG-X-Z-NH_2

(Ac = acetyl, X = tetraethylene glycol linker (CAS 557756-85-1), Z = N-ε-acryloyl-L-lysyl)

$C_{183}H_{300}N_{46}O_{66}$

Monoisotopic mass: 4198.2 Da

Molecular weight: 4200.7 g mol^{-1}

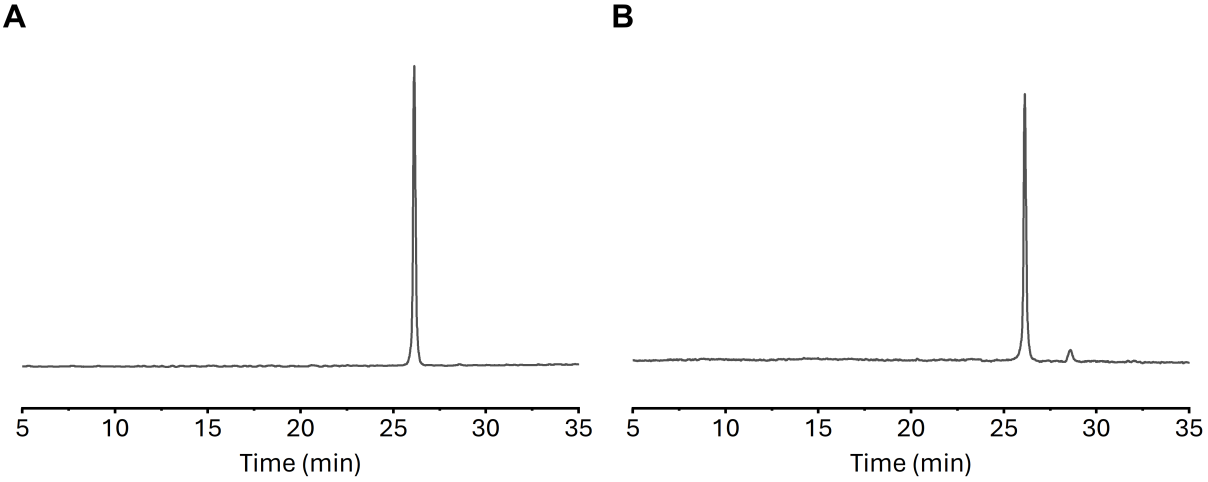

Figure S 16: *Analytical HPLC traces of pure* ***4a*** *(20-50% A in B).* ***(A)*** *Chromatogram at 220 nm.* ***(B)*** *Chromatogram at 280 nm.*

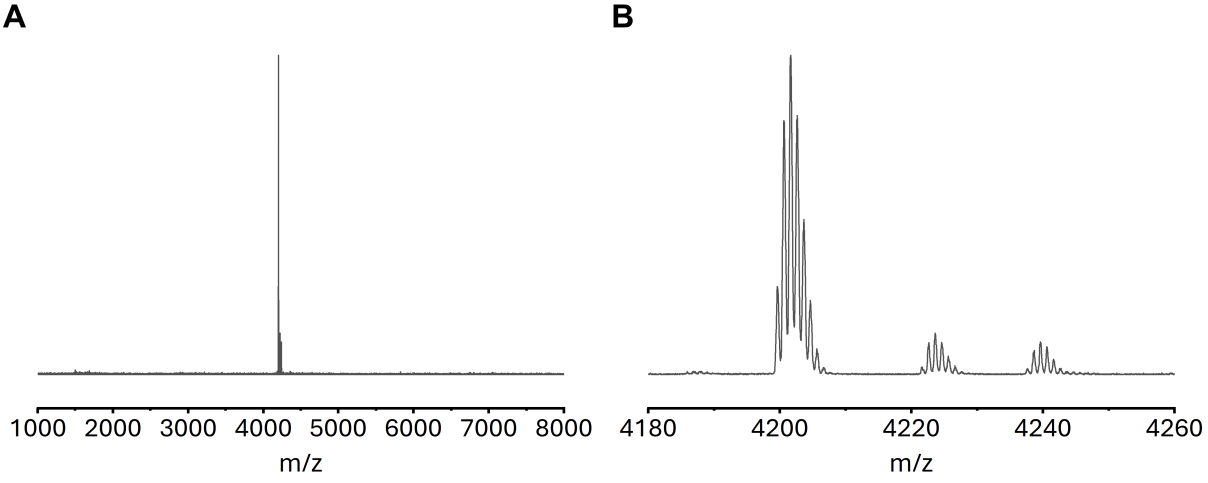

Figure S 17: *MALDI-TOF MS spectra of* ***4a*** *(RP1000-8000 mode).* ***(A)*** *Full spectrum.* ***(B)*** *Zoom in on product isotope pattern. Peaks: $[M+H]^+$ calc. 4199.2 m/z, found 4199.7 m/z; $[M+Na]^+$ calc. 4221.2 m/z, found 4221.6 m/z; $[M+K]^+$ calc. 4237.3 m/z, found 4237.6 m/z.*

Peptide 4b

Z-X-SPEDENAALEEKIAQLKQKNAALKEEIQALEYG-NH_2

(X = tetraethylene glycol linker (CAS 557756-85-1), Z = Cy5.5-yl)

$C_{210}H_{323}N_{46}O_{62}$

Monoisotopic mass: 4481.4 Da

Molecular weight: 4484.2 g mol^{-1}

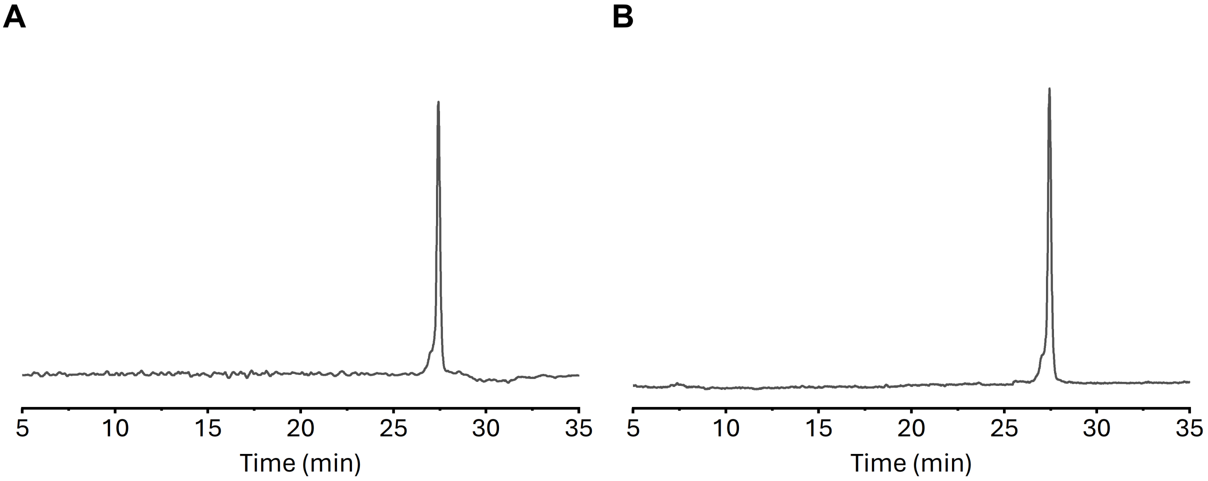

Figure S 18: *Analytical HPLC traces of pure* ***4b*** *(30-60% A in B).* ***(A)*** *Chromatogram at 220 nm.* ***(B)*** *Chromatogram at 280 nm.*

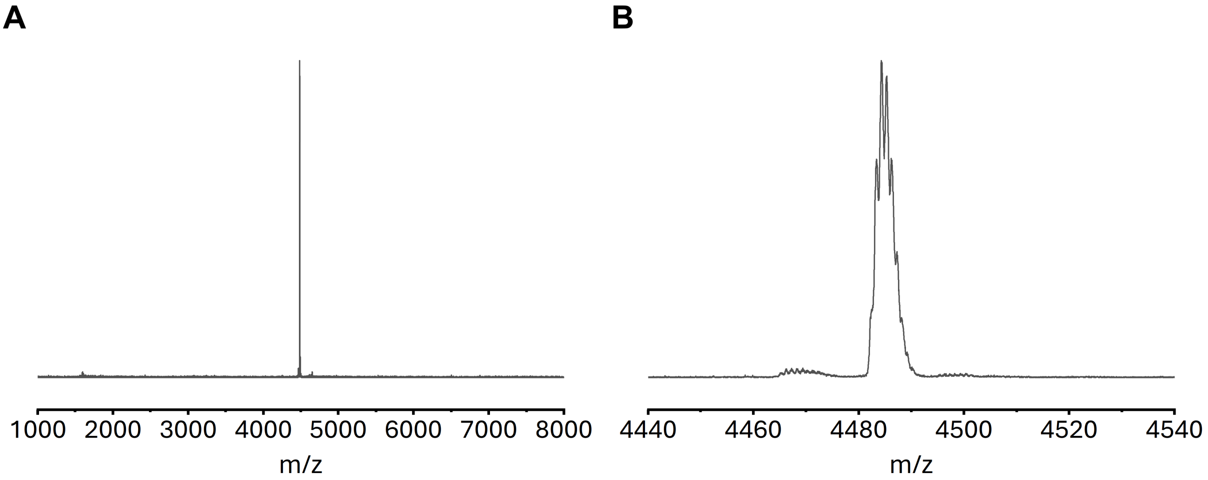

Figure S 19: *MALDI-TOF MS spectra of* ***4b*** *(RP1000-8000 mode).* ***(A)*** *Full spectrum.* ***(B)*** *Zoom in on product isotope pattern. <u>Peaks:</u> $[M+H]^+$ calc. 4482.4 m/z, found 4482.4 m/z.*

Peptide 2b-S

Z-X-EELQAENQAELEIEAEYGQLEPNEALQSEADEI-NH_2

(X = tetraethylene glycol linker (CAS 557756-85-1), Z = 5-/6-carboxytetramethylrhodamine-yl)

$C_{193}H_{285}N_{43}O_{74}$

Monoisotopic mass: 4389.0 Da

Molecular weight: 4391.6 g mol^{-1}

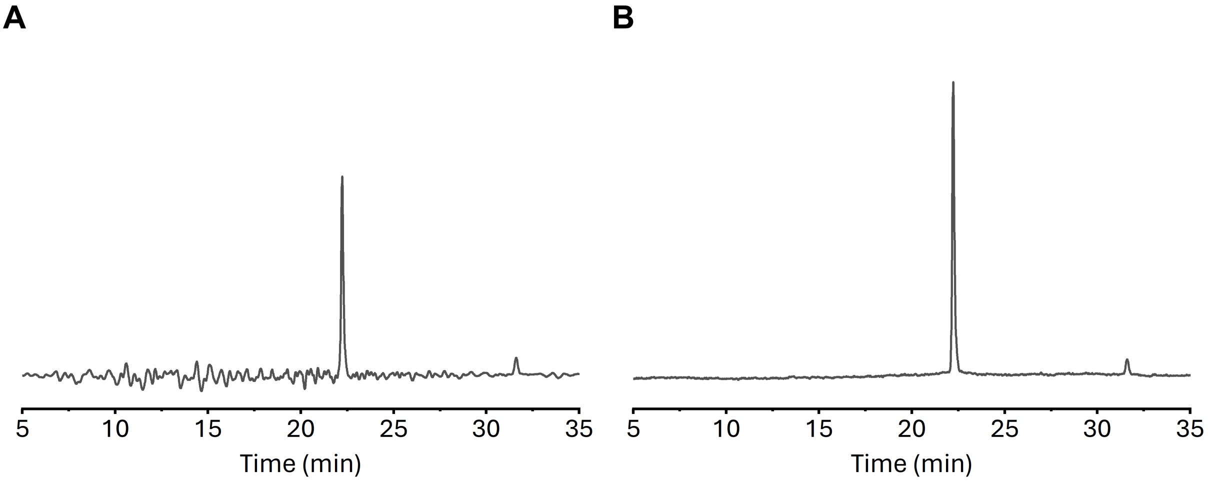

Figure S 20: *Analytical HPLC traces of pure* ***2b-S*** *(20-60% A in B).* ***(A)*** *Chromatogram at 220 nm.* ***(B)*** *Chromatogram at 280 nm.*

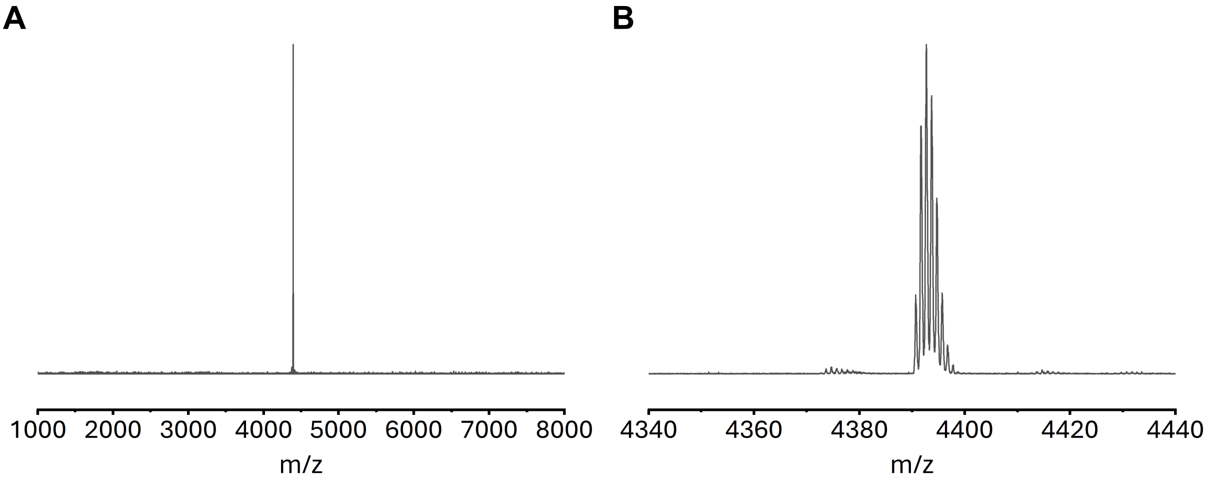

Figure S 21: *MALDI-TOF MS spectra of* ***2b-S*** *(RP1000-8000 mode).* ***(A)*** *Full spectrum.* ***(B)*** *Zoom in on product isotope pattern. Peaks: $[M+H]^+$ calc. 4390.0 m/z, found 4390.7 m/z.*

Peptide 3b-S

Z-X-QAELEKQALNEEKQIYELQGPAKAELDNQIESK-NH_2

(X = tetraethylene glycol linker (CAS 557756-85-1), Z = 5-/6-carboxyfluorescein-yl)

$C_{195}H_{298}N_{46}O_{69}$

Monoisotopic mass: 4388.1 Da

Molecular weight: 4390.8 g mol^{-1}

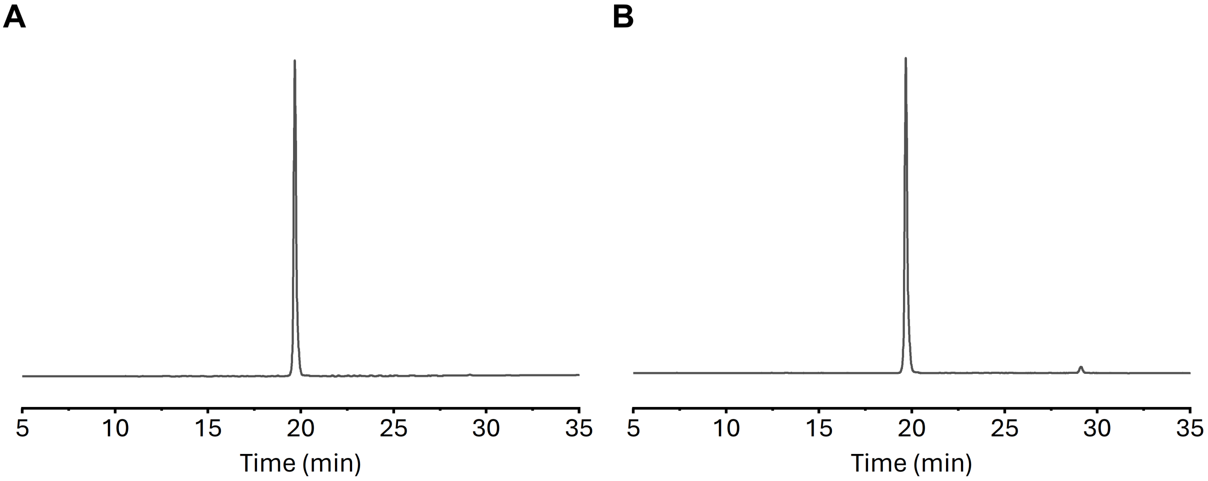

Figure S 22: *Analytical HPLC traces of pure* ***3b-S*** *(20-60% A in B).* ***(A)*** *Chromatogram at 220 nm.* ***(B)*** *Chromatogram at 280 nm.*

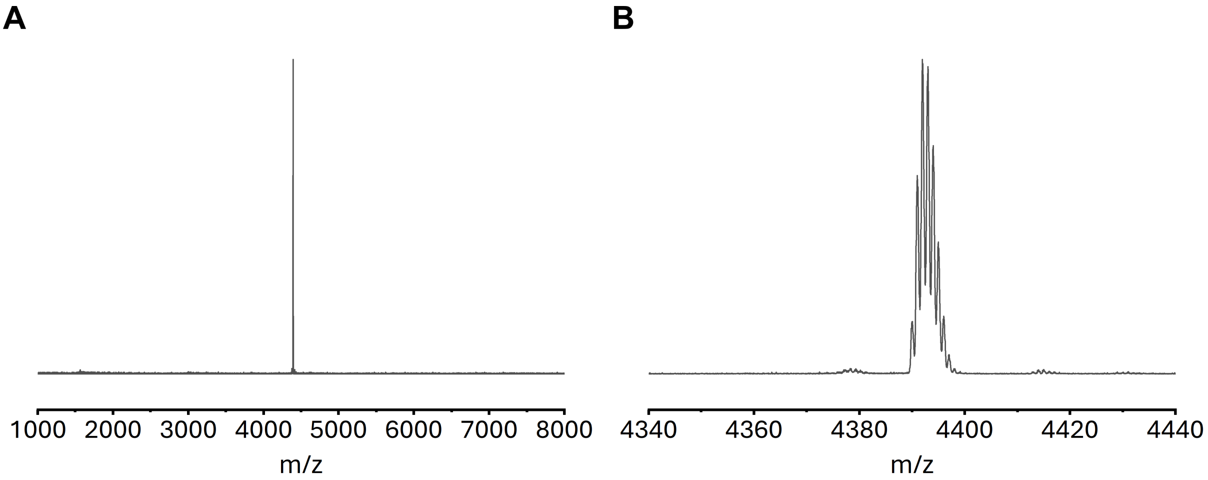

Figure S 23: *MALDI-TOF MS spectra of* ***3b-S*** *(RP1000-8000 mode).* ***(A)*** *Full spectrum.* ***(B)*** *Zoom in on product isotope pattern.* *<u>Peaks:</u>* *$[M+H]^+$ calc. 4389.1 m/z, found 4390.0 m/z.*

Peptide 4b-S

Z-X-AEKQLEEANLQAKELDIAEYGKSPALQKNEEAI-NH_2

(X = tetraethylene glycol linker (CAS 557756-85-1), Z = Cy5.5-yl)

$C_{210}H_{323}N_{46}O_{62}$

Monoisotopic mass: 4481.4 Da

Molecular weight: 4484.2 g mol^{-1}

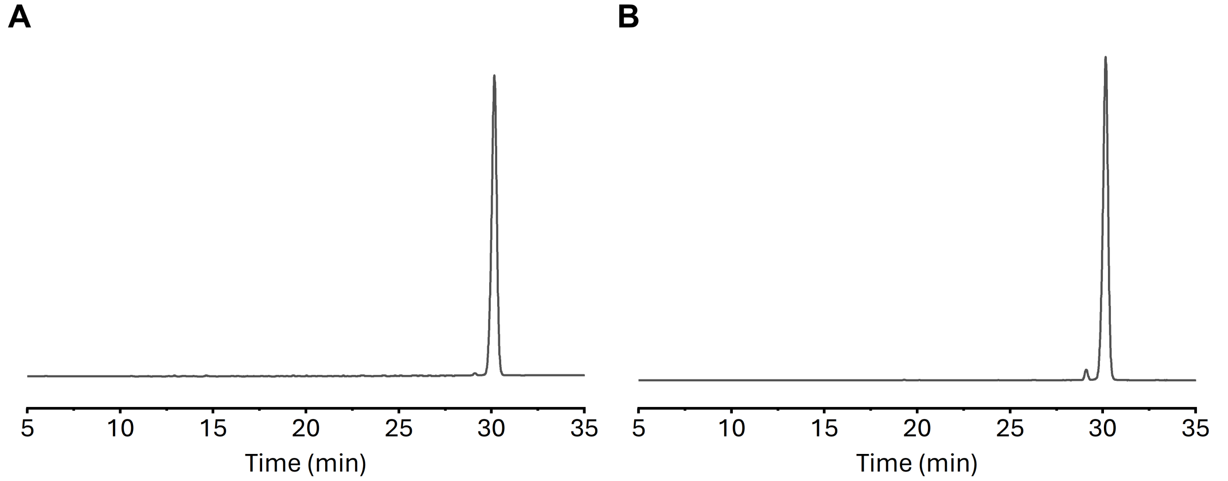

Figure S 24: *Analytical HPLC traces of pure **4b-S** (20-60% A in B). **(A)** Chromatogram at 220 nm. **(B)** Chromatogram at 280 nm.*

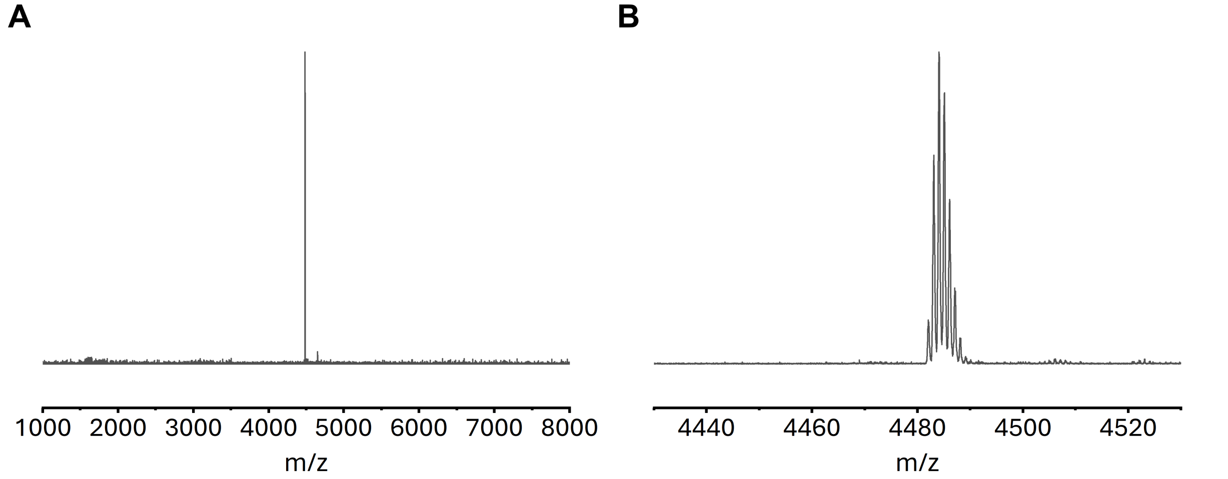

Figure S 25: *MALDI-TOF MS spectra of **4b-S** (RP1000-8000 mode). **(A)** Full spectrum. **(B)** Zoom in on product isotope pattern. Peaks: $[M+H]^+$ calc. 4482.4 m/z, found 4482.0 m/z.*

6 Fluorescence microscopy

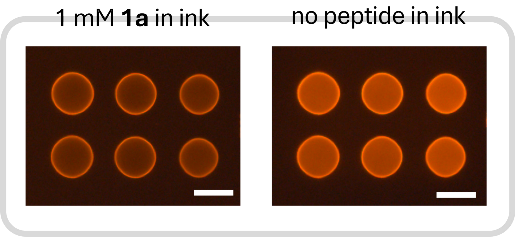

Figure S 26: Capture tests with initial tested ink formulations (without AAm addition). *Peptide **1a** solubility limit in the ink at around 1 mM. Printed structures did not show any capture properties, with very limited diffusion of target peptide **1b** into material. Reference prints without any **1a** in ink showed similar behavior.*

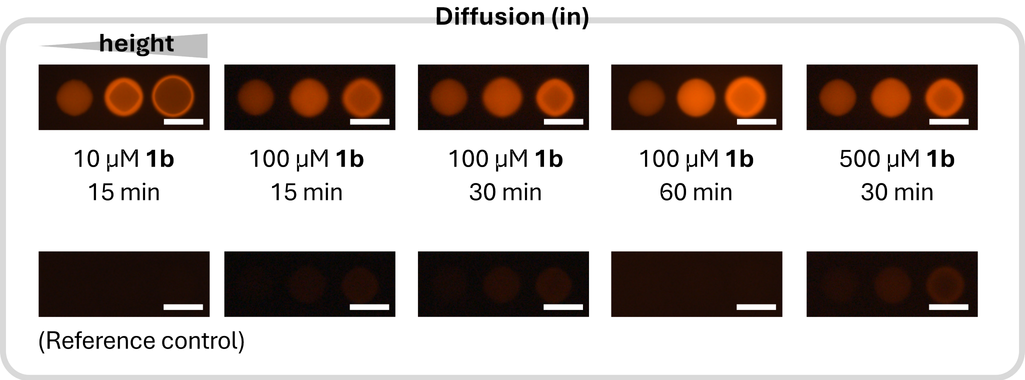

Figure S 27: Fluorescence images of target peptide diffusion into printed structures. (excitation 555 nm; scale bar = 50 µm) *Diffusion of target peptide **1b** into printed cylinders of 4, 7, and 10 µm height under different capture conditions. Second condition was chosen for optimized procedure providing reliable and fast loading of the printed structures with target peptide.*

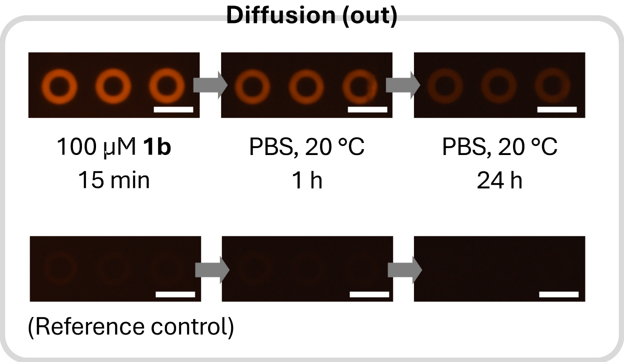

Figure S 28: Fluorescence images of background diffusion of target peptide 1b out of printed structures. (excitation 555 nm; scale bar = 50 µm) *Experiments performed at room temperature in PBS (pH 7.4). Moderate release within one hour was observed, near-complete release within one day.*

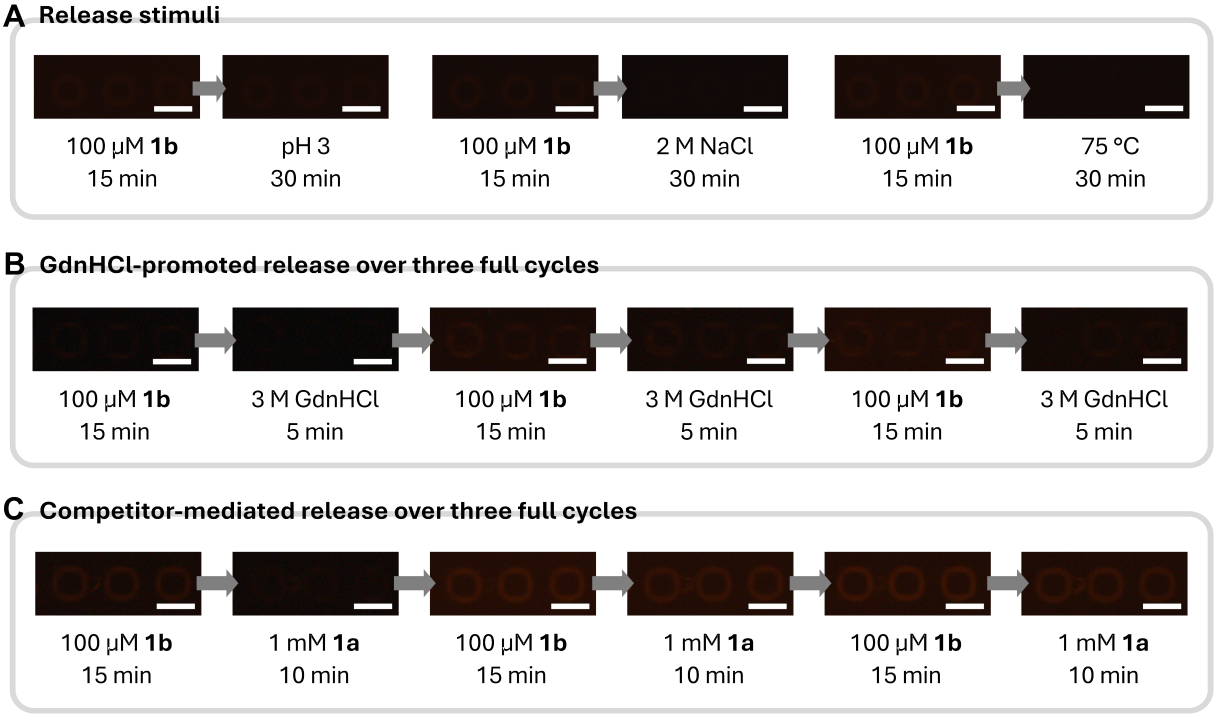

Figure S 29: Fluorescence images from release condition investigations with 3D printed microstructures NOT containing any peptide (= corresponding reference control prints, see Figure 2). (excitation 555 nm; scale bar = 50 µm) (A) *Release studies with different stimuli (pH, ionic strength, temperature).* ***(B)*** *Investigation of cycle stability during repeated capture and release with denaturing agent guanidine hydrochloride (GdnHCl).* ***(C)*** *Selective release studies by addition of competitor* ***1a*** *in solution onto the printed structures, including successful cycle stability test.*

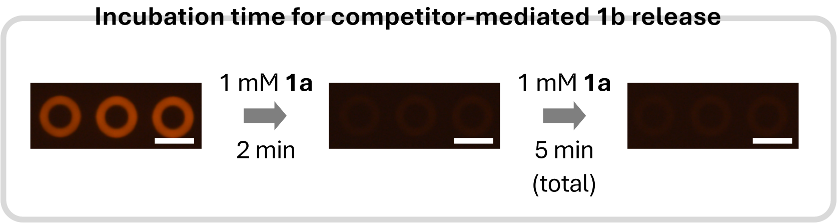

Figure S 30: Fluorescence images for determination of competitor-mediated release kinetics. (excitation 555 nm; scale bar = 50 µm) *Peptide* ***1b*** *was found to be completely released from printed structure at first possible point of observation (2 min after competitor addition). After 5 min, no additional decrease in fluorescence was observed.*

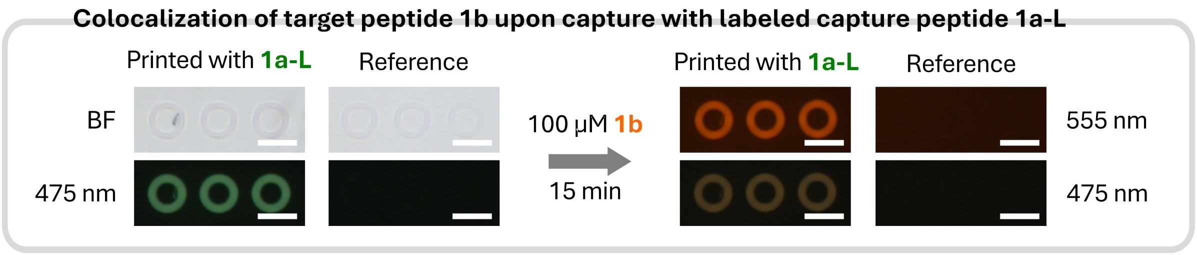

Figure S 31: Fluorescence images of microstructures printed from ink containing labeled capture peptide 1a-L. (scale bar = 50 µm) *Homogeneous covalent incorporation of **1a-L** was visualized with respective excitation of FAM label. Colocalization of **1a-L** and **1b** upon **1b** capture was shown via visualization of the two peptides in the respecetive fluorescence channels. **1a-L** fluorescence was found to be largely decreased after **1b** capture. This was expected due to FRET-mediated fluorescence intensity loss, further indicating colocalization of **1a-L** and **1b** peptides as desired.*

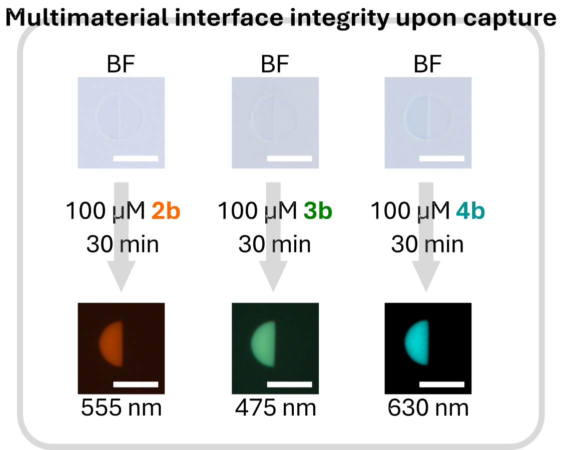

Figure S 32: Fluorescence images of multimaterial prints with half-moon architecture. (50 µm diameter, 6 µm height, scale bar = 50 µm) *Interface integrity between printed areas containing captured target peptides **2b**, **3b**, and **4b** respectively, and reference areas containing no peptide could be visualized. Non-specific diffusion or capture of target peptides into non-desirable reference areas could be excluded.*

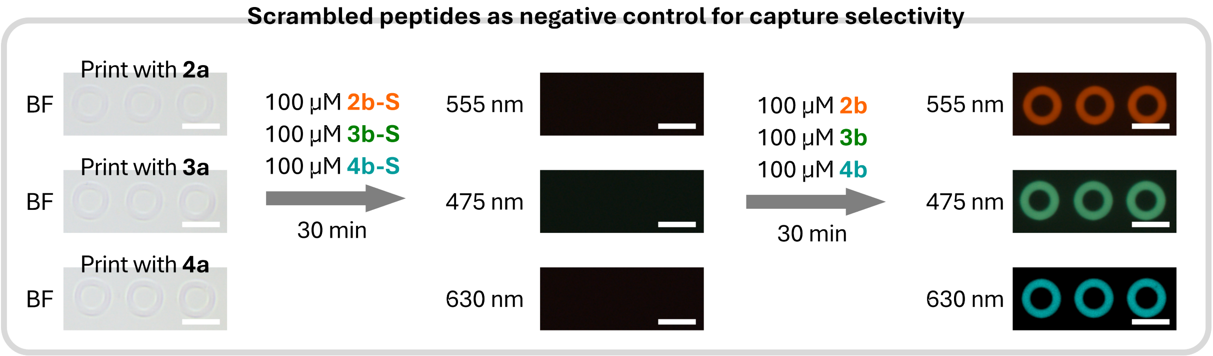

Figure S 33: Fluorescence images of multimaterial prints for scrambled sequence peptide incubation as negative control. (scale bar = 50 µm) *Multimaterial structures containing capture peptides **2a**, **3a**, and **4a** were incubated with scrambled version of the respective target peptides (**2b-S**, **3b-S**, and **4b-S**). No presence of scrambled peptides in printed structures was observed, confirming the coiled-coil formation mechanism as crucial capture handle. To further confirm functionality of the sample, non-scrambled target peptides **2b**, **3b**, and **4b** were subsequently captured in the designated areas of the printed structures.*

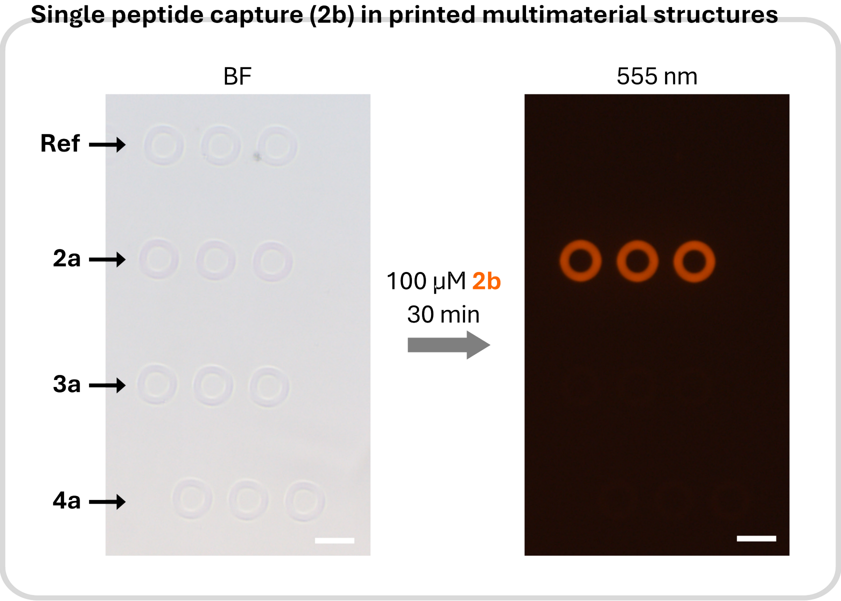

Figure S 34: Fluorescence images of multimaterial structures for selective capture of a single target peptide (scale bar = 50 µm) *Target peptide **2b** was added onto multimaterial prints containing areas for all three orthogonal target peptides. Selective capture in the designated area containing **2a** peptide was confirmed.*

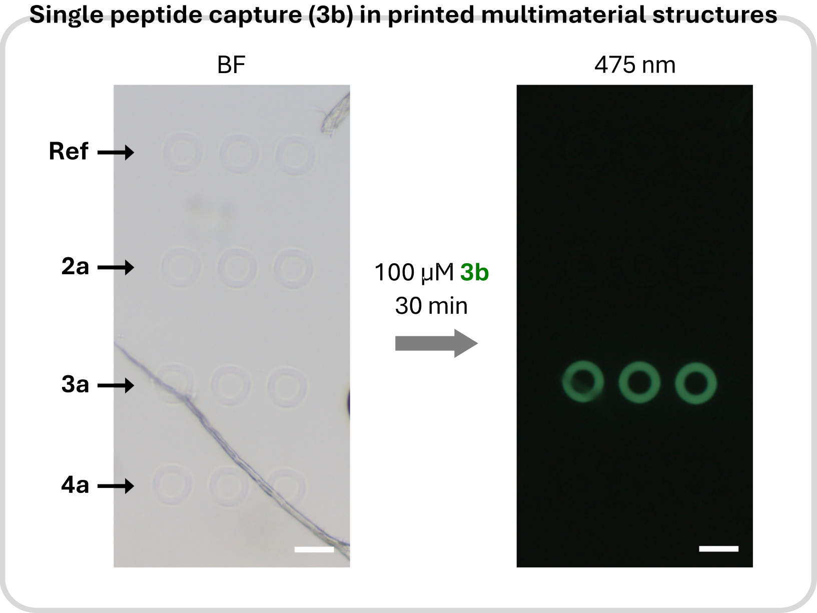

Figure S 35: Fluorescence images of multimaterial structures for selective capture of a single target peptide (scale bar = 50 µm) *Target peptide **3b** was added onto multimaterial prints containing areas for all three orthogonal target peptides. Selective capture in the designated area containing **3a** peptide was confirmed.*

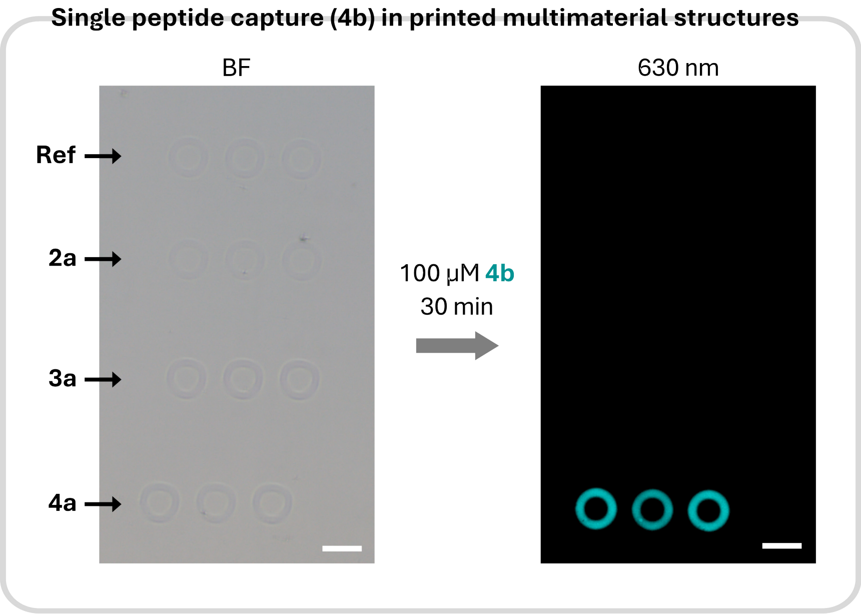

Figure S 36: Fluorescence images of multimaterial structures for selective capture of a single target peptide (scale bar = 50 µm) *Target peptide **4b** was added onto multimaterial prints containing areas for all three orthogonal target peptides. Selective capture in the designated area containing **4a** peptide was confirmed.*

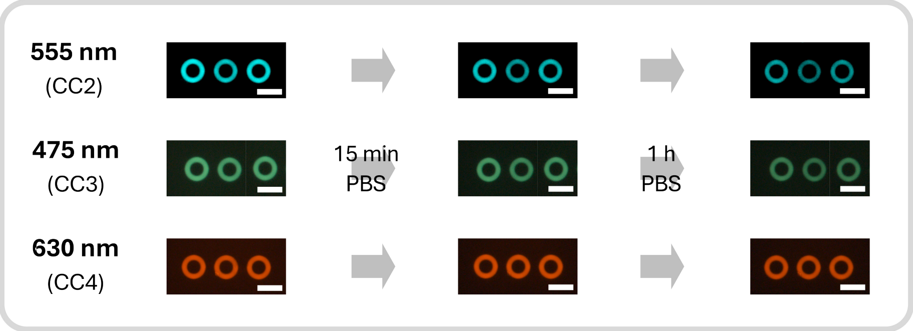

Figure S 37: Background diffusion investigation for multimaterial microstructures in PBS. (scale bar = 50 µm) *Samples were visualized via fluorescence microscopy after 0, 15, and 60 min.*

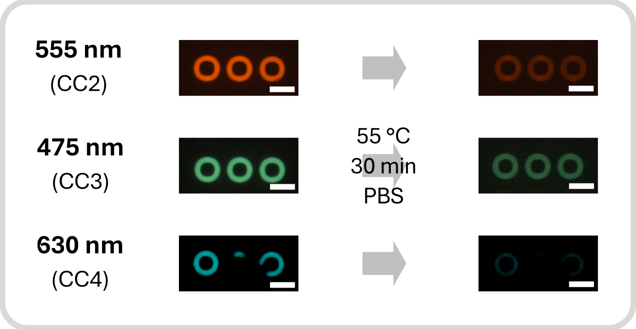

Figure S 38: Heat-mediated target peptide release in multimaterial microstructures. (scale bar = 50 µm) *Equilibration at 55 °C in PBS for 30 min leads to unspecific accelerated release of all target peptides.*

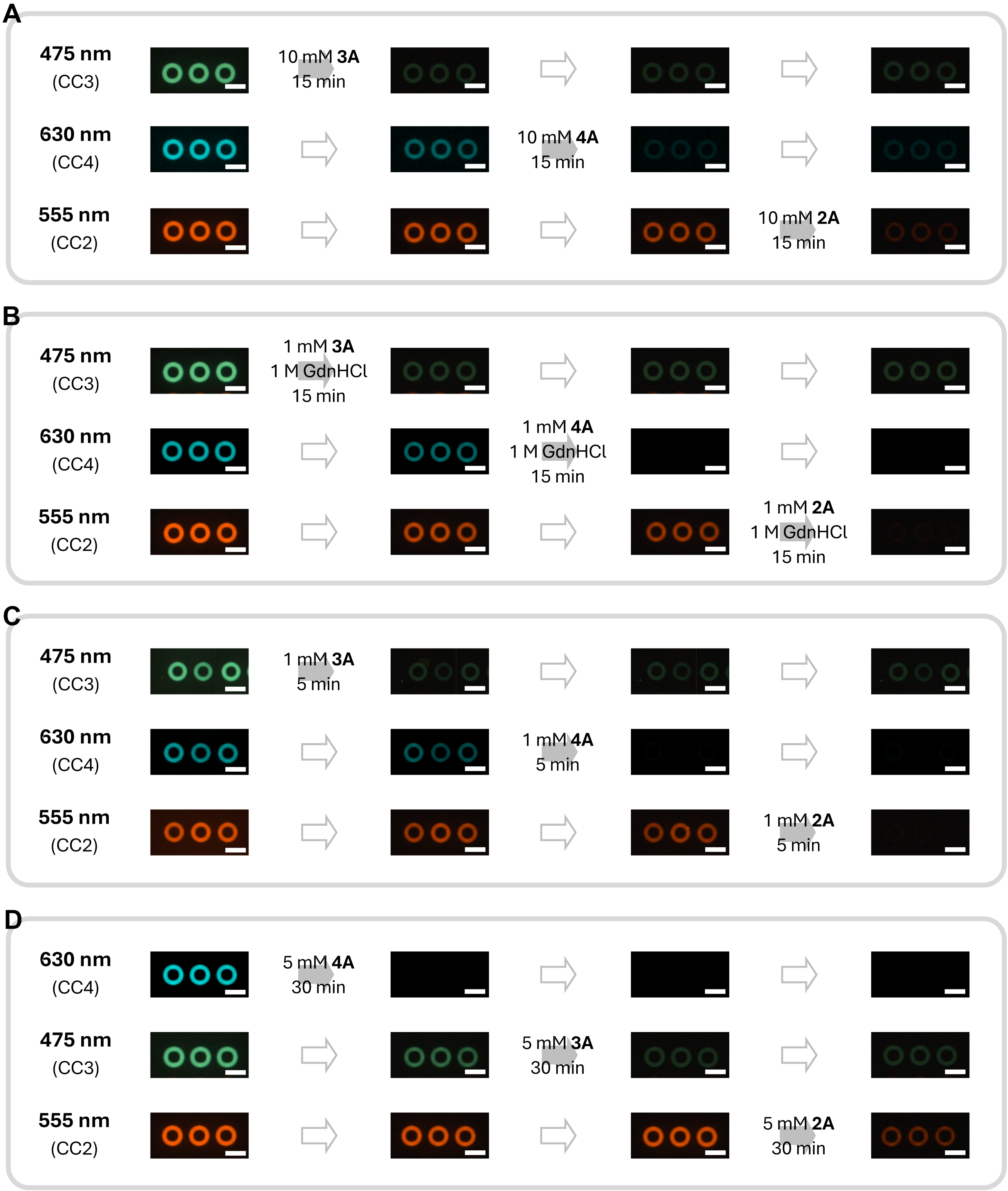

Figure S 39: Release condition optimization for orthogonal target peptide release in multimaterial microstructures. (scale bar = 50 µm) (A) *Subsequent release of peptides **3b**, **4b**, and **2b** via addition of 10 mM competitor solutions for 15 min.* ***(B)*** *Subsequent release of peptides **3b**, **4b**, and **2b** via addition of 1 mM competitor solutions containing 1 M GdnHCl.* ***(C)*** *Subsequent release of peptides **3b**, **4b**, and **2b** via addition of 1 mM competitor solutions for 5 min.* ***(D)*** *Subsequent release of peptides **4b**, **3b**, and **2b** via addition of 5 mM competitor solutions for 30 min.*
